# Targeting the NOTCH1-MYC-CD44 axis in leukemia-initiating cells in T-ALL

**DOI:** 10.1038/s41375-022-01516-1

**Published:** 2022-02-16

**Authors:** Sujan Piya, Yaling Yang, Seemana Bhattacharya, Priyanka Sharma, Huaxian Ma, Hong Mu, Hua He, Vivian Ruvolo, Natalia Baran, R. Eric Davis, Abhinav K. Jain, Marina Konopleava, Hagop Kantarjian, Michael Andreeff, M. James You, Gautam Borthakur

**Affiliations:** 1 Department of Leukemia, Unit 428, The University of Texas MD Anderson Cancer Center, 1515 Holcombe Blvd, Houston, TX 77030 USA; 2 Department of Hematopathology, Unit 72, The University of Texas MD Anderson Cancer Center, 1515 Holcombe Blvd, Houston, TX 77030 USA; 3 Department of Lymphoma/Myeloma, Unit 903, The University of Texas MD Anderson Cancer Center, 1515 Holcombe Blvd, Houston, TX 77030 USA; 4 Department of Epigenetics & Molecular Carcinogenesis, Center for Cancer Epigenetics, Unit 1000, The University of Texas MD Anderson Cancer Center, UTHealth Graduate School of Biomedical Sciences, Houston, TX 77030 USA

**Keywords:** Acute lymphocytic leukaemia, Cell signalling

## Abstract

The NOTCH1-MYC-CD44 axis integrates cell-intrinsic and extrinsic signaling to ensure the persistence of leukemia-initiating cells (LICs) in T-cell acute lymphoblastic leukemia (T-ALL) but a common pathway to target this circuit is poorly defined. Bromodomain-containing protein 4 (BRD4) is implicated to have a role in the transcriptional regulation of oncogenes *MYC* and targets downstream of *NOTCH1*, and here we demonstrate its role in transcriptional regulation of *CD44*. Hence, targeting BRD4 will dismantle the NOTCH1-MYC-CD44 axis. As a proof of concept, degrading BRD4 with proteolysis targeting chimera (PROTAC) ARV-825, prolonged the survival of mice in *Notch1* mutated patient-derived xenograft (PDX) and genetic models (ΔPTEN) of T-ALL. Single-cell proteomics analysis from the PDX model, demonstrated quantitative reduction of LICs (CD34+ CD7+ CD19−) and downregulation of the NOTCH1-MYC-CD44 axis, along with cell cycle, apoptosis and PI3K/Akt pathways. Moreover, secondary transplantation from PDX and ΔPTEN models of T-ALL, confirmed delayed leukemia development and extended survival of mice engrafted with T-ALL from ARV-825 treated mice, providing functional confirmation of depletion of LICs. Hence, BRD4 degradation is a promising LIC-targeting therapy for T-ALL.

## Introduction

Treatment outcomes in patients with relapsed T-cell acute lymphoblastic leukemia (T-ALL) as well as in certain high-risk subgroups of these patients, even with frontline therapy, are dismal and associated with the persistence of leukemia-initiating cells (LICs) [1–3]. Although phenotypic definition of LICs in the T-ALL patients is imprecise, CD34+ CD7+ cells are enriched in LIC compartment [4]. T-ALL LICs are functionally better characterized by high CD44 expression and low reactive oxygen species (ROS) levels [5–7]. Notch1, Myc, and CD44 have been implicated in persistence of LICs in T-ALL [3, 5, 8]. Substantial commonalities exist in pathways activated downstream of NOTCH1 and MYC in T-ALL [9]. Mutated *Notch1* co-occupies the distal enhancer region of the MYC promoting activation of NFkB signaling, *Hes1*, PTEN, and PI3K/Akt pathways in a feed-forward loop circuit that supports leukemia cell growth, proliferation, and self-renewal [8, 10, 11]. Thus, MYC inhibition could represent a powerful therapeutic strategy to treat T-ALL with *Notch1* mutation/activation. It has been difficult to target MYC directly, but MYC can be epigenetically downregulated by the disruption of members of the bromodomain and extra terminal domain (BET) protein family, enriched in large enhancer complexes (termed “super-enhancers”) [12–14]. BRD4, a BET family protein, binds to acetylated lysine residues in histone H3 and provides the scaffold to assemble multi-molecular super-enhancer complexes that drive expression of oncogenes, including Myc and antiapoptotic proteins such as Bcl-2, Bcl-xL, and Mcl-1 [13, 15, 16].

The bone marrow (BM) microenvironment is a protective niche for T-ALL and plays a critical role in chemoresistance and disease persistence [17, 18]. Stromal signaling includes chemokine and adhesion signals by SDF1-α/CXCR4, CD44 and its variants, and other stromal factors [19–21]. CD44, and even more so its variant CD44v8–10, are not only receptors for hyaluronic acid (HA) in the BM stroma but also known to stabilize SLC7A11/_X_CT, a subunit of the cystine-glutamate transporter XC (−) that promotes cystine uptake for glutathione synthesis and mitigates oxidative stress [22–24]. Reactive oxygen species (ROS) mitigation is essential for survival of T-ALL LICs [25], and a strategy to impair ROS mitigation through modulation of expression of CD44 and its variants, could help eliminate LICs.

ARV-825 is a proteolysis-targeting chimera (PROTAC) with three components: a thienodiazepine-based BRD4 ligand, a linker, and a cereblon-binding ligand. This chimera captures a BRD4 molecule and causes its proteasomal degradation via the E3 ligase cereblon. It is then available for degradation of additional BRD4 proteins [24, 26]. Previously, we reported that sustained degradation of BRD4 led to downregulation of CD44, MYC, and CXCR4 in acute myeloid leukemia (AML) stem cells and improved survival in a mouse model of AML [24].

Given the central role of NOTCH1 and MYC in the pathogenesis of T-ALL and the necessity of CD44 and its variant CD44v8–10 for LICs to retain their ‘stemness’ by maintaining a state of low ROS levels, we report here our work with BRD4 degrader in patient-derived xenograft (PDX) and genetic models of T-ALL. We demonstrate that BRD4 regulates CD44 transcription and that degradation of BRD4 dismantles the NOTCH-MYC-CD44 regulatory circuits, depleting T-ALL LICs.

## Materials and methods

### T-ALL cell lines and PDX cells

The human leukemia cell lines CCRF-CEM, HPB-ALL, KOPT-K1, LOUCY, MOLT4, and SUP-T1 were purchased from the ATCC (Manassas, VA, USA) or Deutsche Sammlung von Mikroorganismen und Zellkulturen (Braunschweig, Germany). The mouse T-ALL cell lines LPN228 and LPN49 were generated from conditional knockout mice deficient in PTEN [27]. The cells were maintained in RPMI 1640 medium containing 10% heat-inactivated fetal bovine serum with penicillin and streptomycin (Sigma-Aldrich, St. Louis, MO, USA) at 37 °C with 5% CO_2_ in a humidified incubator. T-ALL PDX cells CUL76, 6506870 and D115 were previously established and kindly provided Dr. Adolfo Ferrando (Columbia University, New York, NY, USA) and Dr. Marina Konopleva’s Lab (UTMDACC, Houston, TX) respectively [28, 29].

### Reagents and antibodies

Reagents and antibodies used in this study are detailed in the Supplemental Methods.

### Real time PCR, immunoblotting and assessment of apoptosis/viability

The methods followed for real time PCR, immunoblotting and assessment of apoptosis/viability via flow cytometry in cells were as previously described [24] and are detailed in the Supplemental Methods.

### ChiP-qPCR, generation of CD44/its variants and BRD4 knockout cells

The method for ChiP-qPCR, generation of ectopic expressed CD44/its variants and BRD4 knockout cells are detailed in Supplemental Methods.

### Cell migration assay, flow cytometry for CXCR4, CD44, and CD98 surface expression and ROS assay

The method for Cell migration assay, flow cytometry for CXCR4, CD44, and CD98 surface expression and ROS assay were as previously described [24] and are detailed in Supplemental Methods.

### In vivo PDXs and ΔPTEN models of T-ALL

Use of all study animals was approved by the MD Anderson Institutional Animal Care and Use Committee under protocol number 00001516-RN00.

CUL76 (Notch1, CDKN2A/B mutation) and D115(Notch1, JAK3 mutation) PDX cells were engrafted into NSG mice and T-cell specific conditional knockout mice that are deficient for Pten in T-lymphoid cells were treated with ARV-825 as described in the Supplemental Methods. The survival of the mice is represented by a Kaplan–Meier plot.

### Single cell mass cytometry (CyTOF) analysis and gene expression analysis

Bone marrow (BM) from D115 PDX mice model and SUP-T1 cells treated with ARV-825 were subjected to single cell mass cytometry (CyTOF) analysis and gene expression analysis respectively as described in the Supplemental Methods.

### Statistical analyses

All data are expressed as the mean ± standard deviation (SD) and representative of triplicate samples. Statistics were generated using GraphPad Prism 7 software. Kaplan–Meier survival analysis was employed to compare the in vivo survival data using log-rank test. Normally distributed groups were compared by two-tailed Student’s *t* test. The statistical significance calculated as *** ≤ 0.001, ** ≤ 0.01, * ≤ 0.05 by a standard Student’s *t* test. In all cases, *P* values ≤ 0.05 were considered statistically significant.

## Results

### BRD4 regulates NOTCH1, MYC, and CD44 expression in T-ALL and CD44 is a direct transcriptional target of BRD4

**Fig. 1:**
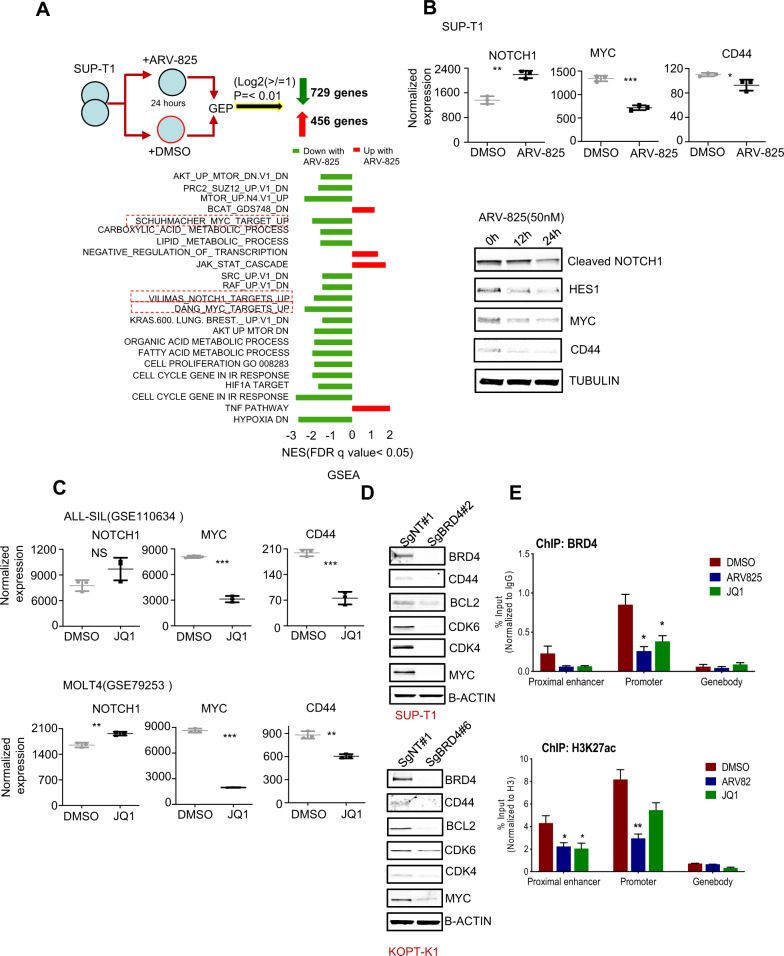
BRD4 regulates NOTCH1, MYC, and CD44 expression in T-ALL and CD44 is a direct transcriptional target of BRD4. SUP-T1 cells were treated with ARV-825 (20 nM) for 24 h. RNA was isolated from the cells and subjected to gene expression profiling (GEP) (*n* = 3). **A** GSEA was performed on Illumina GEP data and revealed high enrichment (normalized enrichment score [NES] > 3, FDR *q* value < 0.05) for several gene sets representing downregulation of Myc and Notch target gene sets along with oncogenic, cell-cycle, hypoxia, and metabolic pathways and upregulation of a gene set of the Wnt/β-catenin and Jak/Stat pathways. **B** Upper panel: Illumina GEP data on SUP-T1 cells treated with ARV-825 for 24 h showed a significant reduction *MYC* and *CD44* while slightly upregulation of *NOTCH1* genes. Lower panel**:** Immunoblots analysis of whole cell lysates (WCL) from SUP-T1 cells treated with ARV-825 (20 nM) for indicated time. **C** Upper panel-GSE110634(ALL-SIL) and lower panel GSE79253(MOLT4) data were curated for *NOTCH1*, *CD44,* and *MYC* genes in ALL-SIL and MOLT4 treated with JQ1 respectively. **D** Immunoblots of WCL of SUP-T1 and KOPT-K1 cells transduced with a nontargeted sgRNA (SgNT) or sgRNA targeting BRD4 (SgBRD4) using CRISPR/Cas9-GFP and then amplified single-cell clone #2 and clone #6, respectively, for different proteins. β-actin was used as a loading control. **E** ChIP-qPCR analysis reveal a significant reduction of BRD4 and H3K27ac occupancy at enhancer and promoter of CD44 in cells treated with ARV825 or JQ1 compared to DMSO control. Error bars represent SD from three different biological replicates (*p < 0.05, **p < 0.01, ***p < 0.001 compared to DMSO).

Our recent studies showed that pharmacological inhibition of BRD4 affects the expression of Myc, CD44, and Notch1 target genes in AML [24]. To obtain a global view of the transcriptional changes in T-ALL with the BRD4 degrader, here we performed genome-wide gene expression profiling (GEP) of SUP-T1 cells after treatment with ARV-825 for 24 h. The treatment resulted in downregulation of 729 genes and upregulation of 456 genes at a significant level (*p* ≤ 0.01) for a onefold change in the log2 value for treatment group. Several genes exhibited much larger fold changes as well (Fig. 1A upper panel). Gene set enrichment analysis (GSEA) using gene signatures from the Molecular Signatures Database highlighted the downregulation of Myc target genes along with gene sets representing other oncogenic pathways: cell-cycle progression, hypoxia response, metabolism, and Notch pathway activity (Fig. 1A bottom panel). Indeed, our gene expression data indicated that Myc and CD44 expression were downregulated upon ARV-825 treatment (Fig. 1B upper panel). Even though, Notch1 expression was partially upregulated, functional cleaved NOTCH1 (NICD) and its direct transcriptional target HES1, along with MYC and CD44 protein expression were decreased upon ARV-825 treatment (Fig. 1B bottom panel). Further, we analyzed NOTCH1, MYC, and CD44 genes in ALL-SIL and MOLT4 cells upon JQ1 (BRD4 inhibitor) treatment from publicly available gene set data GSE110634 [30] and GSE79253 [31] respectively and found that MYC and CD44 are downregulated (Fig. 1C).

To further confirm the association between BRD4 and these targets, we used CRISPR to knock out BRD4 in SUP-T1 and KOPT-K1 T-ALL cell lines (Supplementary Fig. 1A) and observed low expression of Myc and CD44 as well as that of CDK4 and CDK6 (Fig. 1D). BRD4 regulates transcription of Myc and Notch1, but whether BRD4 directly regulates chromatin state and CD44 expression is not known. To address this, we leveraged previously published ChIP-Seq data for BRD4 and histone marks at CD44 at KOPT-K1 [32] and identified high K4me1, K27ac and BRD4 peaks at the enhancer, ~16 kb upstream of CD44 and high K4me3, K27ac, and BRD4 peaks at the promoter (Supplementary Fig. S1B). Enrichment of these histone marks is strongly associated with BRD4 -driven transcription. We performed ChIP-qPCR to validate BRD4-chromatin interactions at these loci in SUP-T1 cells (Fig. 1E and Supplementary Fig. S1B, C) and found that BRD4 was enriched both at enhancer and promoter of CD44 compared to CD44 gene body, an area of little to no enrichment (negative control) (Fig. 1E). In parallel, our ChIP-qPCR analyses revealed a strong enrichment of: H3K4me1 and H3K27ac (associated with active enhancer) on CD44 enhancer, H3K4me3 and H3K27ac (associated with active promoter) on CD44 promoter, and lack of H3K27me3 (associated with gene repression) at both enhancer and promoter compared to CD44 gene body (Fig. 1E and Supplementary Fig. S1B, C). Collectively, these results suggest that the CD44 gene is actively transcribed in SUP-T1 cells. Treatment with BRD4 degrader ARV-825 and small molecule inhibitor JQ1 reduced enrichment of BRD4, H3K4me1, H3K27ac and H3K4me3 on CD44 loci (Fig. 1E and Supplementary Fig. S1C). Taken together, these findings suggest that CD44 is a direct transcriptional target of BRD4.

### BRD4 regulates CD44/CD44v8-10 expression and modulates oxidative stress and oxidative phosphorylation in T-ALL

**Fig. 2:**
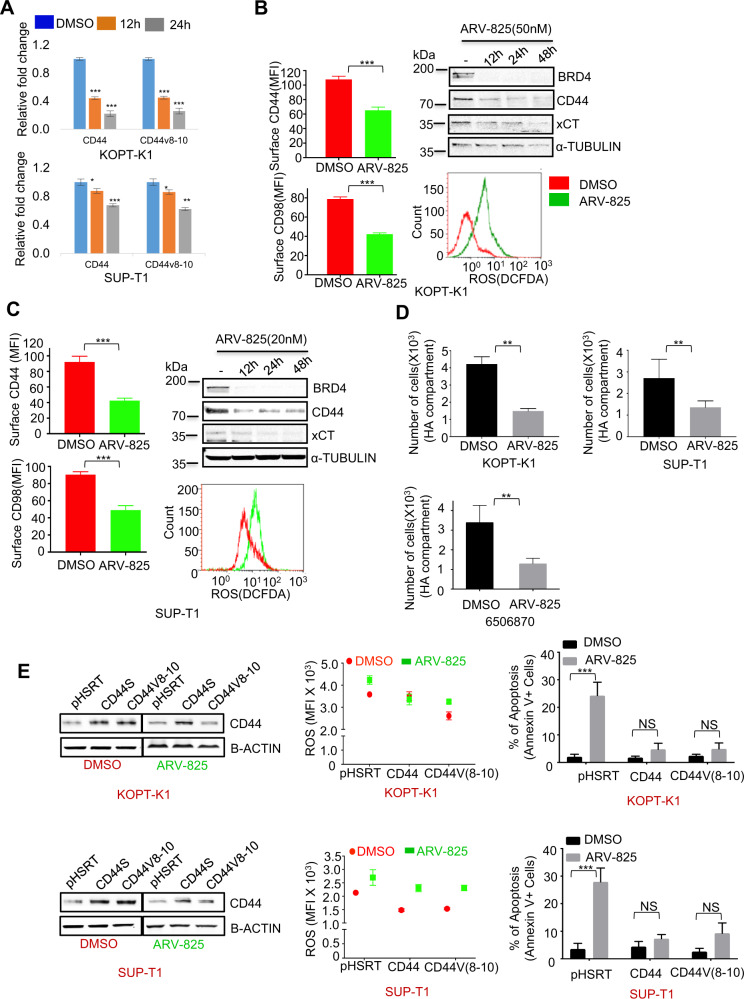
BRD4 regulates CD44/CD44v8-10 expression and modulates oxidative stress and oxidative phosphorylation in T-ALL. KOPT-K1 and SUP-T1 cell lines were treated with ARV-825 (50 nM for KOPT-K1 and 20 nM for SUP-T1) for 0, 12, and 24 h. **A** RNA was extracted from cells, and qPCR analysis of CD44 and CD44v8-10 was performed. Gene expression was normalized to the corresponding 18 S rRNA expression level (*n* = 3). **B** KOPT-K1 (**C**) SUP-T1 cell treated with ARV-825 (50 nM) or 20 nM) as above for 24 h and cells were subjected to flow cytometry analysis of surface expression of CD44(left top panel) and CD98(Left bottom panel), as well, (Right top panel)-WCL of duplicate samples cells treated with ARV-825 for 24 h subjected to immunoblots of whole-cell lysates of KOPT-K1 and SUP-T1 cells with the indicated antibodies. (Right bottom panel) results of an assay performed to determine the level of total ROS generation by KOPT-K1 (**B**) and SUP-T1 (**C**) cells using an ENZ-51011 kit. **D** KOPT-K1, SUP-T1, and T-ALL PDX (6506870) treated with ARV-825 in 50 nM, 20 nM, and 100 nM for 24 h respectively subjected to a migration assay 4 h after incubation in media containing HA (150 ng). **E** CD44- and CD44v8-10–overexpressing KOPT-K1 and SUP-T1 cells (left top panel and bottom panel respectively) treated with ARV-825 (50 or 20 nM) for 24 and subjected to immunoblots with the indicated antibodies and duplicate samples were assayed total ROS (middle panel). Similar samples with 72 h treatment subjected to measure levels of apoptosis (right panel) using flow cytometry. Error bars represent SD from three different biological replicates (*p < 0.05, **p < 0.01, ***p < 0.001 compared to DMSO.

In the context of AML, we showed that CD44 and its oncogenic variant CD44v8-10 are downregulated upon BRD4 degradation and this impairs cellular redox balance [24]. Similar to those findings, we found that BRD4 degradation by ARV-825 treatment resulted in pronounced downregulation of CD44 and CD44v8-10 mRNA in KOPT-K1 and SUP-T1 cells (Fig. 2A). Because CD44 and its variants stabilize SLC7A11/_x_CT, a membrane cystine-glutamate antiporter that maintains low ROS levels in cells, we functionally correlated CD44 downregulation with oxidative stress. As shown in Fig. 2B, C (left), and Supplementary Fig. S2A degradation of BRD4 resulted in reduced surface expression of CD44, CD98, and _x_CT in KOPT-K1 and SUP-T1 cells. It also reduced their total protein levels (Fig. 2B and C, right top panel) and, as a functional correlate, increased ROS levels (Fig. 2B and C, right bottom panel) in KOPT-K1 and SUP-T1 cells. Similarly, treatment with ARV-825 reduced the surface expression of CD44 and increased ROS levels in LPN228 and LPN49, cell lines derived from mouse T-ALL (Fig. S2B).

The importance of adhesive interaction between CD44 and HA to maintain LICs in the BM niche has been confirmed using an anti-CD44 antibody, soluble HA, or hyaluronidase [33, 34]. Therefore, we hypothesized that reduction of surface CD44 expression by ARV-825 treatment would affect the migration of T-ALL cells toward HA. Indeed, this treatment impaired migration of KOPT-K1, SUP-T1, and T-ALL PDX cells to the HA compartment (Fig. 2D).

To further confirm the role of CD44 and CD44v8-10 in oxidative stress in the context of BRD4 degradation, we induced their overexpression in KOPT-K1 and SUP-T1 cells (Fig. 2E left upper and lower panel) respectively. Indeed, overexpression of CD44 and CD44v8-10 partially restored the low ROS status (Fig. 2E middle panel and Supplementary Fig. S2C) and reversed the proapoptotic effect of ARV-825 (Fig. 2E right panel). These results strongly suggest that BRD4 degradation can impair the role of CD44 and its variant in mitigating oxidative stress in T-ALL cells. Interestingly, when we exposed T-ALL cells treated with ARV-825 to the exogenous ROS scavenger N-acetyl-L-cysteine (NAC), it resulted in partial abrogation of ROS generation with substantially reduced apoptosis, indicating that oxidative injury contributes to antileukemic activity of ARV-825 (Supplementary Fig. S2D (top and middle panel).

Finally, to confirm the role of CD44 and CD44v8-10 in migration of cell toward HA, we further extend our experiment with KOPT-K1 and SUP-T1 cells overexpressing CD44 and CD44v8-10. In fact, ARV-825 partly rescue the impaired migration of KOPT-K1 and SUP-T1 cells to the HA compartment (Supplementary Fig. S2D bottom panel).

As c-Myc is critical for the metabolic demands of cancer cells, we tested the impact of BRD4 degradation on oxidative phosphorylation and observed a reduction in oxidative phosphorylation (Supplementary Fig. S2E). Hence, BRD4 degradation modulates T-ALL cell-intrinsic metabolism, ROS mitigation, and cell-extrinsic homing properties.

Since BRD4 degradation impacts Notch1, Myc, CD44, cellular ROS, and cell homing (migration); all of which are critical for T-ALL biology and LIC maintenance, we hypothesized that ARV-825 would be effective as a single agent in treating T-ALL. Treatment of a diverse array of Notch1-mutated human and mouse T-ALL cells with ARV-825 inhibited proliferation and induced apoptosis in a dose-dependent manner, reflected by decreased absolute cell numbers and increased annexin V staining (Supplementary Fig. S2G, H). The calculated inhibitory concentrations ranged from 14 to 125 nM as IC50 values, in a diverse array of T-ALL cells (Supplementary Fig. S2F). Importantly, in PDX-derived T-ALL cells with activating Notch1 mutations (TET2 mut, U2AF1 mut, WT1 mut), ARV-825 inhibited proliferation and induced apoptosis in bulk as well as in the CD34+ CD7+ LIC subset (Supplementary Fig. 2I).

### BRD4 modulates Notch1, Myc, CD44, and PI3K/AKT signaling and apoptosis in T-ALL cells

**Fig. 3:**
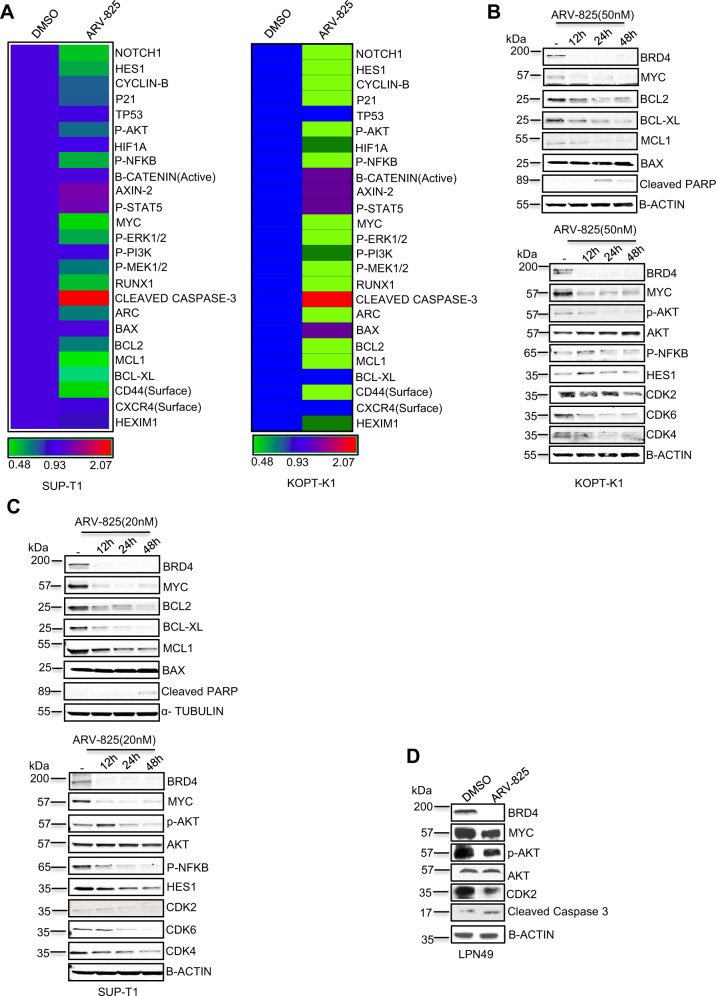
BRD4 modulates Notch1, Myc, CD44, and PI3K/AKT signaling and apoptosis in T-ALL cells. **A** SUP-T1 and KOPT-K1 cells treated with ARV-825 (20 nM and 50 nM) for 24 h and subjected to mass spectrometry-based flow cytometry (CyTOF). The heat map was generated using Prism software (version 8; GraphPad Software, San Diego, CA, USA). **B** KOPT-K1, **C** SUP-T1 and mouse (**D**) T-ALL LPN49 cells treated with ARV-825 in concentration 50 or 20 nM or 15 nM respectively for 24 h. Whole-cell lysates were analyzed using the indicated antibodies. β-actin was used as a loading control. β-actin used as a loading control.

We performed CyTOF analysis to simultaneously profile the status of multiple proteins associated with cell differentiation, survival, proliferation, metabolism, and homing and cell-cycle progression after treatment with ARV-825. In SUP-T1 cells, apart from downregulation of the known BRD4 targets like Myc, Bcl-2, Bcl-XL, and Mcl-1, treatment with ARV-825 substantially decreased the expression of key molecules involved in leukemia persistence, such as HES1 (a direct target of Notch1), PI3K/AKT pathway proteins, and CD44 (Fig. 3A left panel). We observed a similar CyTOF signature in KOPT-K1 cells (Fig. 3A right panel). To validate these findings, we performed immunoblotting with KOPT-K1 and SUP-T1 cells (NOTCH1 mutation) and LPN49 cells (PTEN^−/−^, NOTCH1 mutation). Treatment with ARV-825 reduced the expression of Hes1, p-Akt, Cdk2/4/6, and Myc as well as the antiapoptotic proteins Bcl2, Mcl1, and Bcl-XL, resulting in increased cleaved PARP or caspase-3 expression at 24 and 48 h (Fig. 3B–D). These results confirmed that BRD4 modulates Notch1, Myc, CD44, and PI3K/AKT signaling and apoptosis in T-ALL cells.

### ARV-825 has single-agent antileukemic activity and improves survival in mice with T-ALL and human T-ALL PDX

**Fig. 4:**
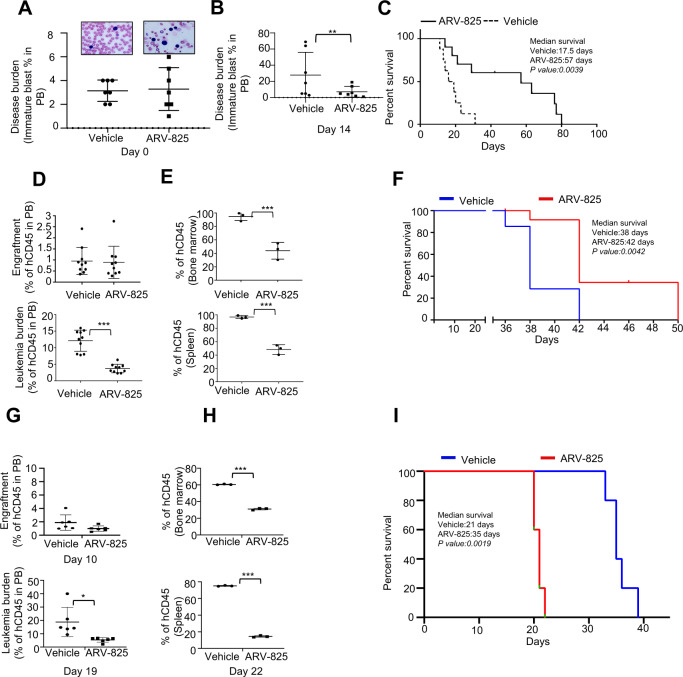
ARV-825 has single-agent antileukemic activity and improves survival in mice with T-ALL. **A** 8-week-old Pten-deficient mice with T-ALL as indicated by the appearance of immature blasts in peripheral blood consider as day 0 and randomly assigned to treatment with a vehicle or ARV-825 (20 mg/kg intraperitoneally twice a week) (*n* = 8). **B** After 14 days of treatment, reduction of disease burden in ARV-825 compared to vehicle group documented as reduced immature blast in blood. **C** Kaplan–Meier survival curve for mice with T-ALL treated with ARV-825 or a vehicle (*p* = 0.0089). **D** Six-week-old NSG mice were implanted with D115 T-ALL PDX cells (1 × 10^6^) through the tail vein (*N* = 10). Leukemia engraftment was confirmed on day 8 via detection of hCD45+ cells in peripheral blood (upper panel) and randomly assigned to treatment with either ARV-825(10 mg/kg). The reduced leukemia burden in ARV-825 treated mice compare to vehicle as seen via flow cytometric analysis of hCD45 in peripheral blood on day 35(lower panel). **E** On day 38, reduced leukemia burden as expression of hCD45 on bone marrow and spleen in ARV-825 treated mice compare to vehicle. **F** Kaplan–Meier survival curve for mice with T-ALL treated with a vehicle or ARV-825 (*p* = 0.0042). **G** NSG mice were implanted with CUL76 T-ALL PDX cells and monitored for 10 days (*n* = 6). Peripheral blood of mice was subjected to flow cytometry analysis to check expression of hCD45 to document engraftment of leukemia and then treated with vehicle, ARV-825(5 mg/kg) or vehicle as above (upper panel). The reduced leukemia burden in ARV-825 treated mice compare to vehicle as seen via flow cytometric analysis of hCD45 in peripheral blood on day 19 (lower panel). **H** On day 22, reduced leukemia burden as expression of hCD45 on bone marrow and spleen in ARV-825 treated mice compare to vehicle. **I** Kaplan–Meier plot of the in vivo activity of ARV-825 against CUL76 PDX engrafted with NSG mice. Significance between ARV-825-treated vs. vehicle-treated mice was determined by a Mantel–Cox Rank Sum test. *P* values < 0.05 were considered to be significant.

To test the therapeutic potential of BRD4 degradation for T-ALL, we used conditional knockout mice with T cell-specific PTEN deletion in which T-ALL developed and that had activating Notch1 mutations [35]. T-ALL establishment was confirmed by the appearance of immature blasts in the peripheral blood of mice (Fig. 4A) and was followed by administration of ARV-825 (20 mg/kg intraperitoneally twice a week. On day 14, ARV-825–treated mice had fewer circulating blasts than did the vehicle-treated mice, confirming the antileukemic effect of this agent (Fig. 4B and Supplementary Fig. S3A). Furthermore, we saw splenomegaly and thymomegaly associated with tissue infiltration of leukemic cells in vehicle treated control mice but normal organ and architecture in ARV-825–treated mice (Supplementary Fig. S3B) on day 28. The antileukemic activity of ARV-825 resulted in markedly longer survival of ARV-825–treated mice than of vehicle-treated control mice (Fig. 4C median survival, 57 days vs. 17.5 days; *p* = 0.0039).

We further validated the antileukemic activity of ARV-825 in a human PDX model of T-ALL. Immunodeficient NSG mice transplanted with D115 human T-ALL PDX cells (activating Notch1 mutations) were treated with ARV-825 (10 mg/kg intraperitoneally three times a week) or a vehicle on day 8 after confirmation of disease establishment (Fig. 4D upper panel). We confirmed reduction of the disease burden in ARV-825 treated mice via flow cytometric analysis of hCD45 in peripheral blood on day 35 (Fig. 4D bottom panel). We further demonstrated reduced hCD45+ cells in BM and spleens in ARV-825 treated mice compare to moribund mice from vehicle group on day 38 (Fig. 4E). Indeed, histological examination of the BM and spleens from mice treated with ARV-825 revealed lower leukemia cell infiltration than in the vehicle group (Supplementary Fig. S3C top panel). Finally, in this aggressive T-ALL PDX model, mice in the ARV-825 cohort had significantly longer survival than did mice in the vehicle cohort (Fig. 4F; median survival time, 42 days vs. 38 days; *p* = 0.0042). Indeed, above findings were confirmed in a repeat experiment using the same PDX model (*n* = 8, median survival 37 days vs. 31, *p* = 0.0004) (Supplementary Fig. S3C-bottom panel).

To generalize the antileukemic activity of ARV-825, we further extended the in vivo study on a human CUL76 PDX (CDKN2A/B mut, Notch1 HD /PEST mut) model of T-ALL. After establishment of disease in NSG mice transplanted with CUL76 cells on day 10 (Fig. 4G upper panel), we administered ARV-825 (5 mg/kg intraperitoneally three times a week) or a vehicle. As above, ARV-825 treated mice had lower leukemia burden by flow cytometric analysis of hCD45 in peripheral blood on day 19 (Fig. 4G bottom panel) and by reduced hCD45+ cells in BM and spleens on day 22 (Fig. 4H). While control mice showed splenomegaly, ARV-825–treated mice had normal organ weight and lower tumor burden by histological examination of the BM and spleens (Supplementary Fig. S3D). Finally, in this aggressive T-ALL PDX model, mice in the ARV-825 cohort had significantly longer survival than did mice in the vehicle cohort (Fig. 4I; median survival time, 21 days vs. 35 days; *p* = 0.0019).

### Disruption of the Notch1-Myc-CD44 axis impairs LIC function and disease progression in mice with T-ALL

**Fig. 5:**
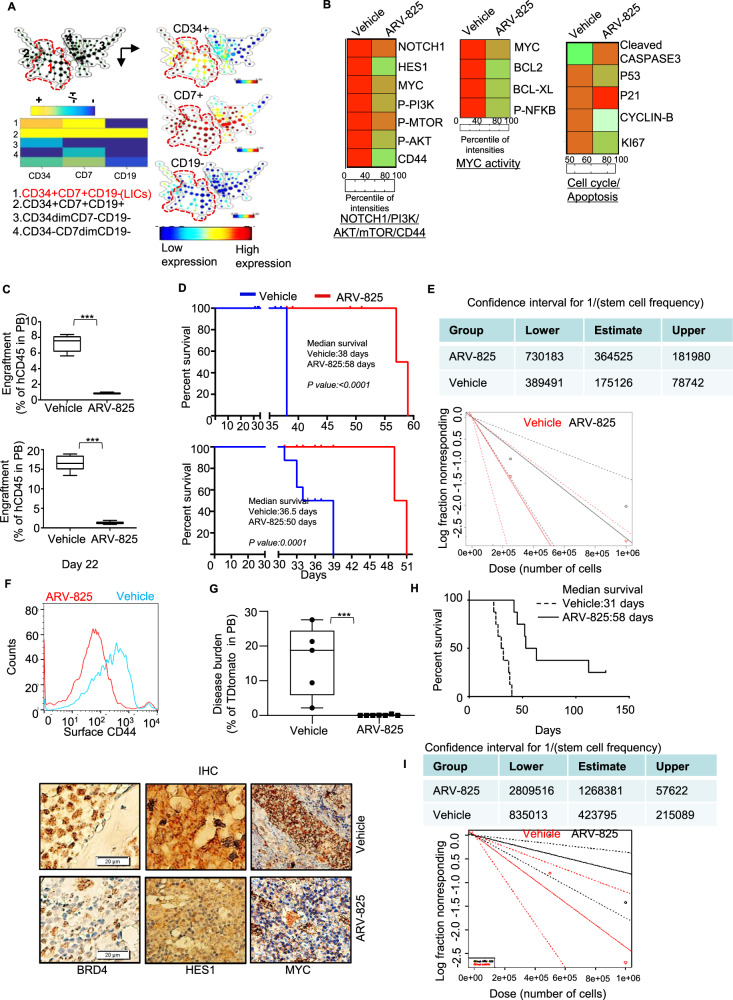
Disruption of the Notch1-Myc-CD44 axis impairs LIC function and disease progression in mice with T-ALL. BM cells were collected from vehicle- and ARV-825–treated mice with D115 T-ALL PDXs on day 38 from above experiment in Fig. 4 and (**A**) subjected to CyTOF, and the resulting data were analyzed using SPADE (version 3.0). The spanning tree was generated according to the expression of CD34, CD7, and CD19. **B** Expression of proteins related to the Notch pathway, Myc activity, the PI3K/AKT/mTOR pathway, cell cycle/apoptosis, and the tumor microenvironment in BM cells from vehicle- and ARV-825–treated mice was determined and quantified in LICs (CD34+ CD7+ CD19− subset; cluster 1). The heat maps were generated using Prism software (version 8) based on the intensities of proteins in vehicle and -treated mice. **C** Duplicate BM cells from mice treated with ARV-825 or a vehicle from the experiment in Fig. 4 were transplanted into NSG mice at two dilutions (0.25 and 1 million cells). The graphs compare the leukemia burden in peripheral blood at day 22 on mice as lower dose (upper panel) and higher dose (lower panel) (**D**) corresponding Kaplan–Meier survival curves for mice with the indicated numbers of cells. **E** LIC frequencies in NSG mice according to limiting dilution transplantation analyses. The dotted lines indicate 95% CIs. **F** BM cells were collected from vehicle- and ARV-825–treated conditional Pten-deficient T-ALL mice on day 28. The bone marrow from above experiment (Fig. 4) in the conditional Pten-deficient T-ALL mouse model which exhibited reduced surface expression of the surface CD44 (top panel) by flow cytometry and also reduced expression of BRD4, HES1, and MYC in ARV-825 treated group in immunohistochemical (IHC) staining (Bottom panel). Duplicate of the same from those experiment, 1 × 10^6^ bone marrow cells were transplanted to 4.5 Gy irradiation normal mice and (**G**) after 30 days leukemia burden was measured and exhibited reduced circulating immature blast counts in mice given ARV-825. **H** Kaplan–Meier survival curve for the secondarily transplanted mice with T-ALL treated with ARV-825 or a vehicle (*p* < 0.0001). **I** LIC frequencies in the mice in the secondary transplantation analyses. The dotted lines indicate 95% CIs.

The persistence of LICs drives relapse and therapy resistance of T-ALL. We performed single-cell proteomic analysis with CyTOF and Spanning-tree Progression Analysis of Density-normalized Events (SPADE; version 3.0) to study the expression of cell-surface and intracellular proteins in rare phenotypically defined subpopulations of T-ALL, including LICs, in BM samples of D115 PDX model from the above experiment. We clustered cell populations from BM hierarchically according to the expression of surface markers, and we displayed them in a single minimal spanning tree, where nodes can be annotated for further analysis as described previously [24]. The CD34+ CD7+ CD19− LIC subset clustered as a single node (cluster 1) in the tree (Fig. 5A left top panel). The expression of individual surface markers as well as intracellular proteins of interest in the SPADE tree for the mouse BM cell populations are presented in Fig. 5A. A heat map of the protein expression in LICs generated from a clustering tree with a single node demonstrated downregulation of NOTCH1-PI3K/AKT-mammalian target of rapamycin (mTOR), CD44, and Myc in LIC nodes in ARV-825–treated mice (Fig. 5B, left and middle). In addition, proteins associated with cell-cycle progression and apoptosis were downregulated in these LICs while cyclin dependent kinase inhibitor, p21 was upregulated (Fig. 5B, right panel). Quantitatively, we observed a lower LIC number in ARV-825–treated mice (*n* = 38135) than in vehicle-treated mice (*n* = 66752).

To functionally validate the quantitative and qualitative impact of BRD4 degradation on T-ALL LICs, we performed serial transplantation from the experiment above using the D115 PDX model (Supplementary Fig. S4A). We transplanted equal numbers of FACS-sorted human cells from the BM of mice given ARV-825 or vehicle treated mice (0.25 × 10^6^ or 1 × 10^6^ hCD45-sorted cells, day 38), into NSG mice and monitored them for disease development and progression and overall survival without any further treatment. Mice that received either cell dose from ARV-825–treated donors had substantially lower circulating hCD45+ cell numbers in peripheral blood than did recipients of ALL cells from vehicle-treated mice (Fig. 5C, top and bottom). The mice injected with cells from the ARV-825–treated group also had considerably longer survival than did their vehicle-treated counterparts, with a median survival time of 58 days vs. 38 days (*p* = 0.0001) and of 50.0 days vs. 36.5 days (*p*- = 0.0001) in mice injected with 0.25 × 10^6^ and 1 × 10^6^ cells, respectively (Fig. 5D, top and bottom). Secondary transplantation of BM cells under limiting dilution conditions (10^4^ to 10^6^ cells/mouse) into secondary transplant recipients revealed an LIC frequency of 1 in 175126 cells for vehicle-treated mice and 1 in 364525 cells(*p* = 0.054) ARV-825–treated mice (Fig. 5E, Table 1). Mice in the vehicle group in secondary transplantation showed higher burdens of leukemic CD45+ cells in blood, BM, and the spleen (Table 1).

**Table 1:** Secondary hCD45-populated PDX transplants in NSG mice.

Treatment group	Input PDX cells (*n*)	Penetrance^a^	hCD45 + cells in selected mice at day 39 (%)		Median survival time (days)
			BM	Spleen	
Vehicle	0.25 × 10^4^	6/8	84.50	75.50	38.0
	1.00 × 10^6^	8/8	95.70	82.57	36.5
ARV-825	0.25 × 10^4^	5/8	39.30	55.60	58.0
	1.00 × 10^6^	7/8	51.43	53.40	50.0

Treatment group	Input Mice T-ALL cells (*n*)	Penetrance^b^
Vehicle	5 × 10^5^	3/6
	1.00 × 10^6^	8/8
ARV-825	5 × 10^5^	0/6
	1.00 × 10^6^	6/8

Secondary transplantation of mice -T-ALL- in 5 weeks 4.5 Gy irradiated black mice.

^a^Number of mice with T-ALL/number of implanted mice at day 22.

^b^Number of mice with T-ALL/number of implanted mice at day 128.

In concordance with our cell line data and PDX data, in the conditional Pten-deficient T-ALL mouse model, we observed reduction in the expression of BRD4, surface CD44, HES1 (a direct target of Notch1), and MYC in BM T-ALL cells from the ARV-825-treated mice (Fig. 5F upper and lower panel). Furthermore, we validated impaired LIC function in this model also with secondary transplantation (Supplementary Fig. S4B). Mice that received BM from the ARV-825 treated group exhibited lower numbers of circulating blasts than from the vehicle-treated group on day 30 (Fig. 5G). Finally, mice receiving cells from the ARV-825–treated group had a markedly longer median survival time than did their vehicle-treated counterparts (58 days vs. 31 days, (*p* < 0.0001) (Fig. 5H). Infusion of BM cells into secondary transplant recipients resulted in LIC frequency of 1 in 423795 in vehicle-treated mice and 1 in 1268381 (*p* = 0.04) in ARV-825–treated mice (Fig. 5I, Table 1). These findings confirmed the therapeutic role of BRD4 degradation in targeting in T-ALL LICs.

### ARV-825 synergizes with inhibitors of Wnt/β-catenin and Jak/Stat pathways

**Fig. 6:**
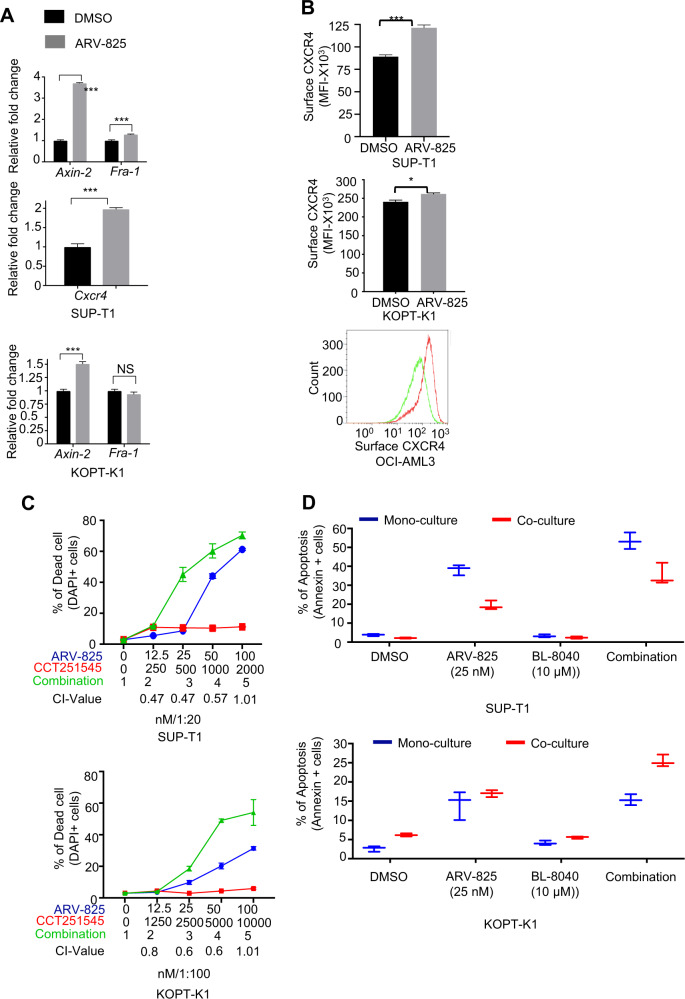
ARV-825 synergize with inhibitor of Wnt/β-catenin and Jak/Stat pathways. SUP-T1, KOPT-K1, and OCIAML3 cells were treated with ARV-825 in concentration 20 nM, 50 nM, and 10 nM respectively for 24 h. **A** qPCR analysis of the *Axin2* and *Fra-1* in three independent samples of SUP-T1 and KOPT-K1*. Cxcr4* in three independent samples KOPT-K1. **B** surface expression of CXCR4 in the SUP-T1, KOPT-K1, and OCIAML3 cells was detected using flow cytometry. **C** and **D** ALL cell lines SUP-T1and KOPT-K1 were cultured with or without mesenchymal stromal cells and treated with ARV-825, CCT251545, or BL-8040 alone or combined for 72 h. The percentage of apoptosis of the cells was calculated using annexin V staining with flow cytometry. Error bars represent SD from three different biological replicates (*p < 0.05, **p < 0.01, ***p < 0.001 compared to DMSO.

Alteration of Wnt/β-catenin and Jak/Stat pathways is important in the pathobiology of T-ALL. Importantly, GSEA revealed upregulation of the Jak/Stat and Wnt/β-catenin pathways with ARV-825 treatment (Fig. 1A). This was validated by qPCR analysis of the Wnt/β-catenin pathway target genes Axin-2 and Fra1 in SUP-T1 and KOPT-K1 cells treated with ARV-825 (Fig. 6A top and bottom panel respectively). In marked contrast with our previous report in AML cells [24], surface expression of CXCR4 was upregulated in both cell lines in response to ARV-825 (Fig. 6B). These findings have translational therapeutic potential, as high surface CXCR4 expression, and Wnt signaling are required for T-ALL LIC activity [36, 37]. It should be noted that unlike in AML CXCR4 is regulated by calcineurin in T-ALL, not by PIM1 kinase [36]. Combination treatment with the Wnt/β-catenin inhibitor CCT251545 or the CXCR4 inhibitor BL-8040 had synergistic effects on apoptosis induction in T-ALL cells when we cultured them either alone (monoculture) or with BM-derived mesenchymal stromal cells (co-culture) to mimic the BM environment (Fig. 6C, D), demonstrating translational potential.

## Discussion

Despite high rates of remission with current frontline therapy, a large proportion of patients with T-ALL, particularly adults, experience relapses with dismal outcomes, with <10% of patients surviving over the long term [1, 9, 38]. LICs represent a reservoir of T-ALL and are believed to drive relapse and treatment resistance of this disease [39]. Notch1, Myc, and CD44 are implicated to have a role in the persistence of these LICs in T-ALL cases [3, 5, 8]. The present study provides evidence that BRD4 is a common therapeutic target that can disrupt the Notch1, Myc, and CD44 pathways to effectively eliminate the T-ALL LICs.

Mutations in the negative regulatory domain of NOTCH1 lead to NOTCH1 activation, and are present in up to 60–70% of patients with T-ALL [40]. In addition, inactivating mutations of FBXW7, which is involved in proteasomal degradation of NOTCH1, are present in 15% of T-ALL cases [9, 41, 42]. Mutated NOTCH1 drives MYC expression and deletion of a copy of the NOTCH-bound MYC enhancer N-Me, extends survival in a NOTCH1-driven T-ALL model [10]. Additional genetic events leading to leukemogenesis in T-ALL cases include loss of the tumor suppressor PTEN; loss of the cell-cycle inhibitors CDKN2A, RB, and CDKN1B; and increased expression of the transcription factors TAL1, LMO1, LMO2, TLX1, and TLX3 and the oncogene MYC [11, 40, 43]. CD44 upregulation also contributes to leukemogenesis and LIC persistence in T-ALL patients [5] and has been identified as a NOTCH1 transcription target. Herein we show that CD44 transcription can be targeted by BRD4 degradation. BRD4 binds to both the promoter and enhancer of CD44, regulating the transcription of CD44 in T-ALL cells. In addition, BRD4 degradation downregulates Myc and active Notch1 (NICD), which are critical to the development of T-ALL.

Hence, the present work confirms that the bromodomain and extraterminal domain family protein BRD4 is a therapeutically actionable transcriptional target in T-ALL with clinically relevant mutations.

Recent therapeutic efforts for T-ALL have relied heavily on targeting mutant Notch1 and its activating events with gamma secretase inhibitors and have been limited by on-target toxic effects [44]. In contrast, our work with the BRD4 degrader ARV-825 is focused on downregulation of NOTCH1 targets and cell-intrinsic pro-survival and/or antiapoptotic proteins as well as interaction with BM microenvironment interactions. Mechanistically, the data from the present study link degradation of BRD4 with transcriptional downregulation of CD44 and its variants, increasing oxidative stress. Using conditional Pten-deficient T-ALL mouse model and NOTCH1-mutated disseminated T-ALL PDX models, which recapitulate several features of human T-ALL biology, we showed that disruption of the NOTCH1-MYC-CD44 axis interferes with the maintenance of leukemia by targeting the LIC population. Indeed, single-cell proteomic analysis of BM cells of ARV-825–treated mice using CyTOF revealed marked downregulation of NOTCH1, MYC, and CD44 along with a significant quantitative decrease in the phenotypically defined LIC population. Furthermore, we demonstrated that reduction in the overall LIC population resulted in extended survival of mice after secondary transplantation. Although the genetic mouse model used in our study has a Pten deletion, these mice often have secondarily acquired activating NOTCH1 mutations [35]. Our findings show the common role of BRD4 in the NOTCH1-, MYC-, and CD44-regulatory axis and we propose that BRD4 is a single target that can be used to disrupt all these pathways simultaneously eliminating T-ALL LICs.

Although we identified BRD4 degradation as a therapeutic means of eliminating T-ALL LICs, our gene array data raise concerns about upregulated CXCR4, other microenvironment niche molecules, and Wnt/β-catenin signaling after BRD4 degradation. These findings may point to potential mechanisms of resistance for bromodomain and extraterminal domain degraders when tested for treatment of T-ALL in clinical trials. Our preliminary work provides guidance toward the use of therapeutic combinations, including inhibition of Wnt/β-catenin signaling or CXCR4 expression to overcome these potential resistance mechanisms (Fig. 6). In our AML study, we showed that BRD4 degradation results in downregulation of surface CXCR4 through downregulation of PIM1 kinase, which phosphorylates CXCR4 [24]. In contrast, CXCR4 expression was not impacted in T-ALL cells, likely because of calcineurin-mediated control of CXCR4 expression in these cells [36].

## Supplementary information

Supplemental materials

Supplementary figures

Supplementary table
