# Supplementary material for: Targeting the NOTCH1-MYC-CD44 axis in leukemia-initiating cells in T-ALL
Source: Leukemia. 2022 Feb 16;36(5):1261–73. doi: 10.1038/s41375-022-01516-1 (PMC9061299; doi:10.1038/s41375-022-01516-1)
Supplement: Supplementary file 1 — Supplemental materials [file 41375_2022_1516_MOESM1_ESM.docx]

**Supplementary materials and methods**

**Reagents and antibodies**

ARV-825 was obtained from Arvinas, Inc. Antibodies against AKT (cs#9272s), p-AKT (cs#4060s), BRD4 (cs#13440/Abcam128874), BAX (cs#2772s), CD44 (cs#37259), CDK2 (cs#2546s), CDK6 (cs#3136s), c-MYC (cs#5605) and cleaved PARP (cs#9541) were obtained from Cell Signaling Technology (Danvers, MA, USA). Antibodies against β-actin (sc-47778), BCL2 (sc-7382), BCL-_XL_ (sc-56021), CDK4 (sc-23896), HES1 (sc-166410), p-NFκB (sc-514451), and α-tubulin (sc-53646) were obtained from Santa Cruz Biotechnology (Dallas, TX, USA). An antibody against MCL-1 (BD 559027) and flow antibodies against CD44-PE (BD560570) and CXCR4-APC (BD 551967) were obtained from BD Biosciences (San Jose, CA, USA). Antibodies against _x_CT-DY488 and N‑Terminus (LS-C142125-100) were obtained from LifeSpan Biosciences (Seattle, WA, USA). An antibody against CD98-APC (#128212) was obtained from BioLegend (San Diego, CA, USA).

**Real-time PCR**

RNA was isolated using a PureLink RNA Mini Kit (Ambion; Thermo Fisher Scientific). The concentration of RNA was measured using a NanoDrop 2000 UV-Vis Spectrophotometer (Thermo Fisher Scientific), and 500 ng of RNA was converted to cDNA using a Verso cDNA Synthesis Kit (Thermo Fisher Scientific). One microliter of the cDNA (equivalent to 10 ng of starting RNA) was used for each real-time PCR in triplicate. Real-time PCR was performed in triplicate using PowerUp SYBR Green Master Mix (Applied Biosystems, Beverly Hills, CA) with a 7900HT Fast Real-Time PCR system as directed by the manufacturer (Applied Biosystems) and subjected to analysis using RQ Manager software (version 1.2.1; Applied Biosystems). Primers were prepared by Integrated DNA Technologies (Coralville, IA, USA) and used at a concentration of 500 nM. Primer sequences are listed in Table S2.

**Immunoblotting**

T-ALL Cells were lysed in RIPA buffer (1% NP-40, 0.5% sodium deoxycholate, 0.1% sodium dodecyl sulfate, 50 mM Tris-Cl, pH 7.5, 150 mM NaCl) in the presence of 1X protease cocktail inhibitor. Soluble lysates were subjected to sodium dodecyl sulfate-polyacrylamide gel electrophoresis and transferred to a polyvinylidene fluoride membrane (Bio-Rad, Hercules, CA, USA). Membranes were probed with specific antibodies. Signals were visualized using an Odyssey Infrared Imaging System (LI-COR Biosciences, Lincoln, NE, USA) and quantitated using Image Studio Lite software (LI-COR Biosciences). β-actin or α-tubulin was used as a loading control.

**Assessment of apoptosis/viability via flow cytometry**

T-ALL cells were incubated with ARV-825 for 72 hours to determine dose response. Apoptosis was analyzed via flow cytometry (Gallios Flow Cytometer; Beckman Coulter, Fullerton, CA, USA) following staining with annexin V-FITC (Thermo Fisher Scientific, Waltham, MA, USA) and DAPI (Invitrogen, Carlsbad, CA, USA). CountBright beads (Molecular Probes, Grand Island, NY, USA) were added resuspension annexin binding buffer to allow for measurement of the absolute number and viable cells. Half-maximal inhibitory concentrations were calculated using CalcuSyn software (BIOSOFT, Cambridge, UK) as described previously(1).

Apoptosis of T-ALL stem/progenitor cells was determined as described previously (2),(1). Briefly, T-ALL PDX cells were incubated with increasing concentrations of ARV-825. After 72 hours, cells were stained with annexin V-APC (#550475), CD34-FITC (#555821), CD19-PE-Cy7(#25019382) and CD7-PE (#340581; BD Biosciences) and then washed and stained with DAPI prior to analysis using flow cytometry.

**ChIP-qPCR**

Chromatin immunoprecipitation (ChIP) assays were performed with modifications to previously described high-throughput ChIP protocol(3),(4). SUP-T1 cells were incubated with or without ARV-825(25 nM) or JQ1(500 nM) for 24 hours, crosslinked in 1% formaldehyde for 10 min at room temperature, followed by 125mM glycine for 5 min to stop crosslinking. Cells were collected and washed with ice cold phosphate-buffered saline and lysed for 30 min on ice in buffer (12 mM Tris-HCl pH 7.5, 6 mM EDTA pH 8.0, 0.5 % SDS) supplemented with protease inhibitors. Lysates were fragmented with a Bioruptor (Diagenode, Denville, NJ, USA) to obtain DNA fragments ranging from 200-600 bp. After centrifugation, supernatants were pre-cleared for 1 hour with Dynabeads Protein G (Invitrogen), and incubated overnight at 4°C with IgG, histone post-translational modifications H3K4me1, H3K27ac, H3K4me3, H3K27me3 and H3 (all from Abcam), and BRD4 (Bethyl Laboratories) antibodies conjugated to Dynabeads Protein G (Invitrogen). The DNA-bound protein complexes were collected next day using Dynamag, washed with low-salt, high-salt and LiCl containing buffers, treated with RNase and Proteinase K, and reverse crosslinked overnight followed by DNA extraction. The DNA region of interest was detected by SYBR green real-time quantitative PCR using primers encompassing BRD4 binding and H3K4me3/H3M27me3 enrichment loci on human CD44 promoter and enhancer determined using ChIP-Seq data in KOPT-K1 cells (GSE54379) (5). The primer sequences used for ChIP-qPCRs are listed in Table S2. A primer set in the CD44 gene body with no apparent histone mark or BRD4 enrichment served as the negative control region for all ChIPs. The BRD4 ChIP was normalized to IgG and calculated as the percent input (chromatin used for ChIP). Similarly, the percent histone mark enrichment was calculated by normalizing first to IgG and then to histone H3 (total histones). Primer sequences are listed in Table S2.

**Generation of BRD4-knockout and CD44 and variants overexpressed cell lines**

BRD4 knockout in T-ALL cell lines (SUP-T1 and KOPT-K1) was carried out following standard procedures. Briefly, CRISPR/Cas9-GFP sgRNA constructs were purchased from Applied Biological Materials (Richmond, British Columbia, Canada). The plasmids pMD2.G and psPAX2 were gifts from Didier Trono (EPFL, Switzerland**.**; Addgene plasmids 12259 and 12260, respectively). The packaging and target sequences containing these plasmids were transfected into HEK293T cells under the manufacturer’s protocol (jetPRIME transfection reagent; Polyplus-transfection SA, New York, NY, USA). Supernatants were collected 48 hours after transfection, filtered, and used to infect T-ALL cells in the presence of hexadimethrine bromide (Polybrene; 8 μg/mL) for 72 hours. Single GFP+ cells grew for 3-4 weeks to generate clones, and target gene knockout was validated via immunoblotting.

The plasmids used to induce overexpression of CD44 and CD44v8-10 with a GFP-tagged pHRST lentiviral vector were provided by Sung-Ho Goh (Precision Medicine Branch, Research Institute, National Cancer Center, Goyang, Gyeonggi-do, Republic of Korea). Overexpressing cell lines were similarly generated via lentivirus-mediated overexpression of CD44 and CD44v8-10 as described previously(6).

**Cell migration assay**

Cell migration assays were carried out using a Corning HTS 24 Transwell system (Sigma-Aldrich, St. Louis, MO, USA) as described previously(1). Briefly, KOPT-K1, SUP-T1, and T-ALL PDX cells were treated with ARV-825 (50, 20, and 50 nM, respectively). After 24 h of incubation, 2 x 10^5^ cells in 200 μL of serum-free media were seeded on the insert cup transwell plate and placed over the receiver wells containing serum-free media with HA (150 ng). Four hours after incubation, cells were collected from the receiver wells and counted using Trypan blue staining with a Beckman Vi-CELL counter.

**CXCR4, CD44, and CD98 surface staining and flow cytometry**

ARV-825–treated T-ALL cell lines were stained as described previously (1). Briefly, cells were incubated with saturating concentrations of a PE- or APC-conjugated monoclonal anti-CD44, anti-CXCR4, or anti-CD98 antibody for 30 min at room temperature. The appropriate isotype-matched antibody was used as a negative control. The surface expression of CD44 and CD98 was analyzed using a Gallios Flow Cytometer.

**Measurement of total ROS levels**

Total ROS levels in T-ALL cells were measured using an ROS-ID Total ROS detection kit as directed by the manufacturer (Enzo Life Sciences, Farmingdale, NY, USA). Briefly, 5 x 10^5^ cells were incubated with ROS detection dye (Oxidative Stress Detection Reagent-Green) for 30 min at 37°C in the dark. Cells were then washed and analyzed using flow cytometry, and the flow cytometric data were assessed using Kaluza software (Beckman Coulter).

**Surface and intracellular staining and CyTOF analysis**

The antibodies used for CyTOF are listed in Table S1. T-ALL cells were incubated with ARV-825 for 24 h (KOPT-K1, SUP-T1 cells were treated with ARV-825 (50, 20 nM, respectively), and 1 x 10^6^ cells were subjected to surface and intracellular staining with metal-conjugated antibodies as described previously(1). Samples were analyzed using a CyTOF mass cytometer fitted with an AS5 autosampler (DVS Sciences, Sunnyvale, CA, USA). The bead signature was routinely applied to normalization of the CyTOF data before analysis. The data were analyzed using FlowJo software (version 10; BD, Franklin Lakes, NJ, USA).

BM cells isolated from mice bearing disseminated T-ALL PDXs at day 38 (vehicle- and ARV-825–treated) were subjected to surface and intracellular staining, and data from Helios were analyzed using SPADE (version 3.0). SPADE tree maps were generated using all surface markers of representative LICs in phenotypically defined cell populations. The boxed annotations were identified by the expression of CD34, CD7, and CD19. The CD34+CD7+CD19- LIC subset as defined in previous studies(2),(7) was located in the SPADE trees as cluster 1 with surface markers of LICs. COLORS SPADE tree according to its colored versions based on the intensities of different proteins was illustrated along with a heat map of expression of proteins generated using Prism software (version 10) based on the percentiles of intensities with respect to treatment with a vehicle, whereas ArcSinh-transformed counts for each protein were illustrated in boxes in CD34+CD7+CD19- LICs. Detailed of Antibodies are listed in Table S1.

***In vivo* PDX model and transgenic T-ALL mouse model**

All animal studies were conducted in accordance with the guidelines approved by the guidelines of the Institutional Animal Care and Use Committees at The University of Texas MD Anderson Cancer Center. Male NOD/SCID/IL-2rgamma null (NSG) mice (6 weeks old; The Jackson Laboratory, Bar Harbor, ME, USA) were intravenously injected with D115 T-ALL PDX cells (1.5 x 10^6^ cells/100 uL PBS). The mice were monitored daily for evidence of leukemia. At day 14, peripheral blood was collected retro-orbitally. Mononuclear cells from blood were stained to assess the leukemic burden according to the number of human CD45+ (hCD45) cells demonstrated by flow cytometry to confirm leukemia engraftment as described previously. The mice were then randomly grouped for treatment with a vehicle or ARV-825 (10 mg/kg intraperitoneally three times a week; n = 10 each) formulated in 5% ethanol, 20% Solutol HS-15 and 75% D5W (5% dextrose in water). On day 35, the leukemia burden was compared in peripheral blood samples from mice in the two treatment groups as described above. On day 38, three moribund mice from vehicle and randomly three mice from ARV-825 treatment groups were killed via CO2 asphyxiation and cervical dislocation, and splenocytes and bone marrow (BM) cells were subjected to quantification of hCD45+ cells. Femur, spleen, and liver samples were collected for staining with hematoxylin and eosin. In parallel, splenocytes and BM cells were subjected to proteomic analysis using cytometry by time of flight (CyTOF). The survival of the mice was analyzed and presented in a Kaplan-Meier curve. The above animal experiment was repeated with minor changes as follow- engraftment confirmation, grouping and treatment start on day 11. The survival of the mice was analyzed and presented in a Kaplan-Meier curve as above.

PDX Cells recovered from bone marrows of mice on day 38 from above experiments was injected in to NSG mice (0.25x106 and 1x10^6^ sorted human CD45+ cells) and mice were followed for disease development and survival as above without any further treatment. On day 22, PB was processed to measure leukemic burden as hCD45+ by flow cytometry as described above. On day 39, one mouse per treatment group was killed, and the penetrance of disease in the BM and spleen was measured

Similarly, CUL76 T-ALL PDX cells (2 x 10^6^ cells/100 uL PBS) were implanted on male NSG mice. The mice were monitored daily for evidence of leukemia. At day 10, peripheral blood was collected retro-orbitally and then leukemia engraftment confirmed as described previously. The mice were then randomly grouped for treatment with a vehicle or ARV-825 (5 mg/kg intraperitoneally three times a week; n = 6 each) formulated as above on day 19, the leukemia burden was compared in peripheral blood samples from mice in the two treatment groups as described above. On day 22, one mouse from both treatment groups were sacrificed and splenocytes and bone marrow (BM) cells were subjected to quantification of hCD45+ cells. Femur, spleen, and liver samples were collected for staining with hematoxylin and eosin. The survival of the mice was analyzed and presented in a Kaplan-Meier curve. Detailed of PDXs are listed in Table S3.

**Conditional Pten-knockout T-ALL mouse model**

The mean disease-free survival of T-cell specific conditional knockout mice that are deficient for Pten in T-lymphoid cells using Lck-Cre transgenic mice and tdTomato is 110 days. The Pten deficient eight-week-old male mice were randomly assigned to either vehicle or ARV-825 (20 mg/kg, intraperitoneally twice a week) (n=8). Before injection (day 0) and day 14 after ARV-825 treatment, leukemic burden was confirmed in peripheral blood. On day 29, leukemia in bone marrow (BM), thymus and spleen were evaluated by H&E morphology, size measurement from some sacrificed mice.

Leukemia cells (1 or 0.5x10^6^ / mouse) collected from spleens of each group from above experiment (day 29) were injected into 5-week-old syngeneic C57/B6 mice after sublethal dose 4.5 Gy irradiated and followed for disease development (n=8). On day 30, leukemic burden as tdTomato positive cells in peripheral blood was measured by flow cytometry. Histologic staining of their tissues was performed at Histology core facility of MDACC. The LIC frequency was calculated using ELDA software (http://bioinf.wehi.edu.au/software/elda/).

**Gene expression profiling**

SUP-T1 cells treated with ARV-825 were harvested after 24 hours. Total RNA was extracted amplified, labeled using *in vitro* transcription, and hybridized to Illumina HT12 (version 4) human whole-genome arrays as described previously(1). The data were deposited into the Gene Expression Omnibus database. The accession number is GSE97707.

**Mice -T-ALL sequencing result:**

LPN49 Exon 34 P2291T A2292G


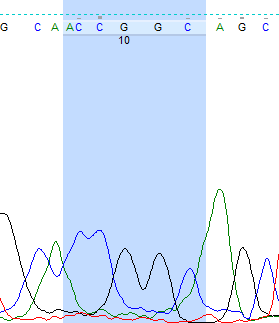

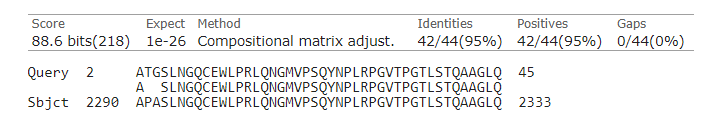


LPN49 Exon34, R2406L**Supplementary figure legends**


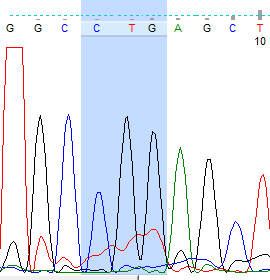

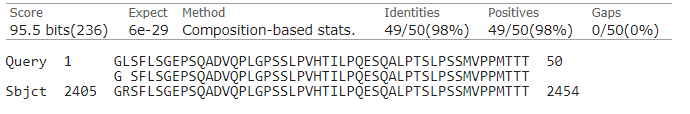


**Fig. 1. CD44 is a direct transcriptional target of BRD4.**

(A) Whole-cell lysates of SUP-T1 and KOPT-K1 cells transduced with a nontargeting sgRNA (SgNT) or sgRNA targeting BRD4 (SgBRD4) with CRISPR/Cas9-GFP and then amplified single-cell clones were subjected to immunoblotting with different proteins. Tubulin was used as a loading control. (B) IGV browser landscapes from ChIP-Seq data in KOPT-K1 cells (GSE54379)(5) showing enrichment of BRD4, H3K4me1, H3K27ac, H3K4me3, H3K27me3 compared to whole cell extract (WCE, input control) along the CD44 loci (top: enhancer, middle: promoter and bottom gene body). The region analyzed by ChIP-qPCR assays is highlighted in the box. (C) ChIP-qPCR analysis reveal a significant reduction of H3K4me1 and H3K4me3 occupancy at enhancer and promoter of CD44 in cells treated with ARV825 or JQ1 compared to DMSO control. No significant enrichment of H3K27me3 was observed compared to negative control gene body region. Error bars represent SEM from three different biological replicates (* p<0.05, ** p<0.01, *** p<0.001 compared to DMSO.

**Fig. 2. BRD4 modulates surface expression of CD44/CD98/ xCT , oxidative stress , oxidative phosphorylation and apoptosis**

(A) KOPT-K1 (upper panel) and SUP-T1 (lower panel) cells were treated with ARV-825 (50 and 20 nM, respectively for 24 h and subjected to flow cytometry for surface CD44, CD98 and _x_CT and (B) LPN228 and LPN49 mouse T-ALL cells were treated with ARV-825 (30 nM) for 24 h and processed for flow cytometric analysis of surface CD44 and total ROS following a standard protocol as described supplemental methods. (C) Histogram of flow cytometry of ROS in CD44- and CD44v8-10–overexpressing KOPT-K1 and SUP-T1 cells treated with ARV-825 (50 or 20 nM respectively) for 24 hours. (D) KOPT-K1 and SUP-T1 cell transduced with vector control pHSRT treated with ARV-825 (50 and 20 nM respectively) with and without N-acetayl-L-cysteine (NAC) (1μM) for 24 hour or 72 hours and processed for flow cytometric analyses of ROS and apoptosis respectively. (Bottom panel) CD44- and CD44v8-10–overexpressing KOPT-K1 and SUP-T1 cells treated with ARV-825 (50 or 20 nM respectively) for 24 hours and then subjected to a migration assay 4 hours after incubation in media containing HA (150 ng). (E) The mitochondrial metabolic activity test was used to obtain bioenergetic parameters for oxidative stress by adding the ATP synthase inhibitor oligomycin A (2 μM) to derive the ATP-linked oxygen consumption rate, FCCP (1.6 μM) to uncouple the mitochondria for maximal oxygen consumption, and antimycin A (0.5 μM) in SUP-T1 cells treated with ARV-825 for 24 h using an extracellular flux analyzer (Seahorse Bioscience, North Billerica, MA, USA).Human cell lines /PDX, and mouse T-ALL cells were treated with ARV-825 at different concentrations for 72 hours. (F) Absolute cell numbers were determined using Trypan blue staining with a Beckman Vi-CELL counter or counting beads with flow cytometry, and half-maximal inhibitory concentrations for ARV-825 were calculated using CalcuSyn software. (G) Absolute numbers and percent apoptosis of KOPT-K1(left panel) and SUP-T1(right panel) and (H) mouse T-ALL cells (LPN228 left panel and LPN49 right pane) treated with ARV-825 at a wide range of concentrations for 72 hours. Counting beads were used to determine the absolute cell numbers, and annexin V/DAPI staining was performed to measure the percent apoptosis with flow cytometry. (I) After 72 hours treatment of ARV-825, 6506870 T-ALLPDX cells were stained with annexin V, CD45, CD34, and CD7, and the absolute cell numbers and percent apoptosis for bulk LICs and a CD34+CD7+ LIC subset were determined using flow cytometry.

**Fig. 3. ARV-825 reduces the leukemia burden in mice**

(A) Eight-week-old Pten-deficient mice with T-ALL treated with either vehicle or ARV-825**.** Numbers of immature blasts in peripheral blood of the mice on day 14 as demonstrated by Giemsa staining. (B) the T-ALL burden in the mice according to organ architecture and hematoxylin and eosin staining on day 22. (C) Top panel: Hematoxylin and eosin stains showing infiltration of leukemia in BM and the spleen in D115 implanted mice from both treatment groups on day 38 **(**Magnification, 60X; scale, 50 μM).Bottom panel: Eight-week-old Pten-deficient mice with T-ALL treated with either vehicle or ARV-825 and follow for survival curve (D) Splenomegaly and Hematoxylin and eosin stains showing infiltration of leukemia in BM and the spleen in CUL76 implanted mice from both treatment groups on day 22 **(**Magnification, 60X; scale, 50 μM).

**Fig. 4.**  **Experimental design for secondary transplantation.**

(A) Six-week-old NSG mice were injected with T-ALL PDX cells as described in supplemental methods in Fig. 5C at a limited dilution (0.25 or 1.00 x 10^6^ cells) through the tail vein and **{**followed as experimental design. (B) Eight-week-old Pten-deficient mice with T-ALL as described in Fig. 5H followed as experimental design for secondary transplantation.

**References**

1. Piya S, Mu H, Bhattacharya S, Lorenzi PL, Davis RE, McQueen T, et al. BETP degradation simultaneously targets acute myelogenous leukemia stem cells and the microenvironment. *The Journal of clinical investigation.* 2019;129(5):1878-94.

2. Gerby B, Clappier E, Armstrong F, Deswarte C, Calvo J, Poglio S, et al. Expression of CD34 and CD7 on human T-cell acute lymphoblastic leukemia discriminates functionally heterogeneous cell populations. *Leukemia.* 2011;25(8):1249-58.

3. Blecher-Gonen R, Barnett-Itzhaki Z, Jaitin D, Amann-Zalcenstein D, Lara-Astiaso D, and Amit I. High-throughput chromatin immunoprecipitation for genome-wide mapping of in vivo protein-DNA interactions and epigenomic states. *Nat Protoc.* 2013;8(3):539-54.

4. Malladi VS, Nagari A, Franco HL, and Kraus WL. Total Functional Score of Enhancer Elements Identifies Lineage-Specific Enhancers That Drive Differentiation of Pancreatic Cells. *Bioinform Biol Insights.* 2020;14:1177932220938063.

5. Knoechel B, Roderick JE, Williamson KE, Zhu J, Lohr JG, Cotton MJ, et al. An epigenetic mechanism of resistance to targeted therapy in T cell acute lymphoblastic leukemia. *Nature genetics.* 2014;46(4):364-70.

6. Choi ES, Kim H, Kim HP, Choi Y, and Goh SH. CD44v8-10 as a potential theranostic biomarker for targeting disseminated cancer cells in advanced gastric cancer. *Sci Rep.* 2017;7(1):4930.

7. Ma W, Gutierrez A, Goff DJ, Geron I, Sadarangani A, Jamieson CA, et al. NOTCH1 signaling promotes human T-cell acute lymphoblastic leukemia initiating cell regeneration in supportive niches. *PloS one.* 2012;7(6):e39725.
