# Supplementary material for: Targeting the NOTCH1-MYC-CD44 axis in leukemia-initiating cells in T-ALL
Source: Leukemia. 2022 Feb 16;36(5):1261–73. doi: 10.1038/s41375-022-01516-1 (PMC9061299; doi:10.1038/s41375-022-01516-1)
Supplement: Supplementary file 2 — Supplementary figures [file 41375_2022_1516_MOESM2_ESM.pptx]

## Slide 1
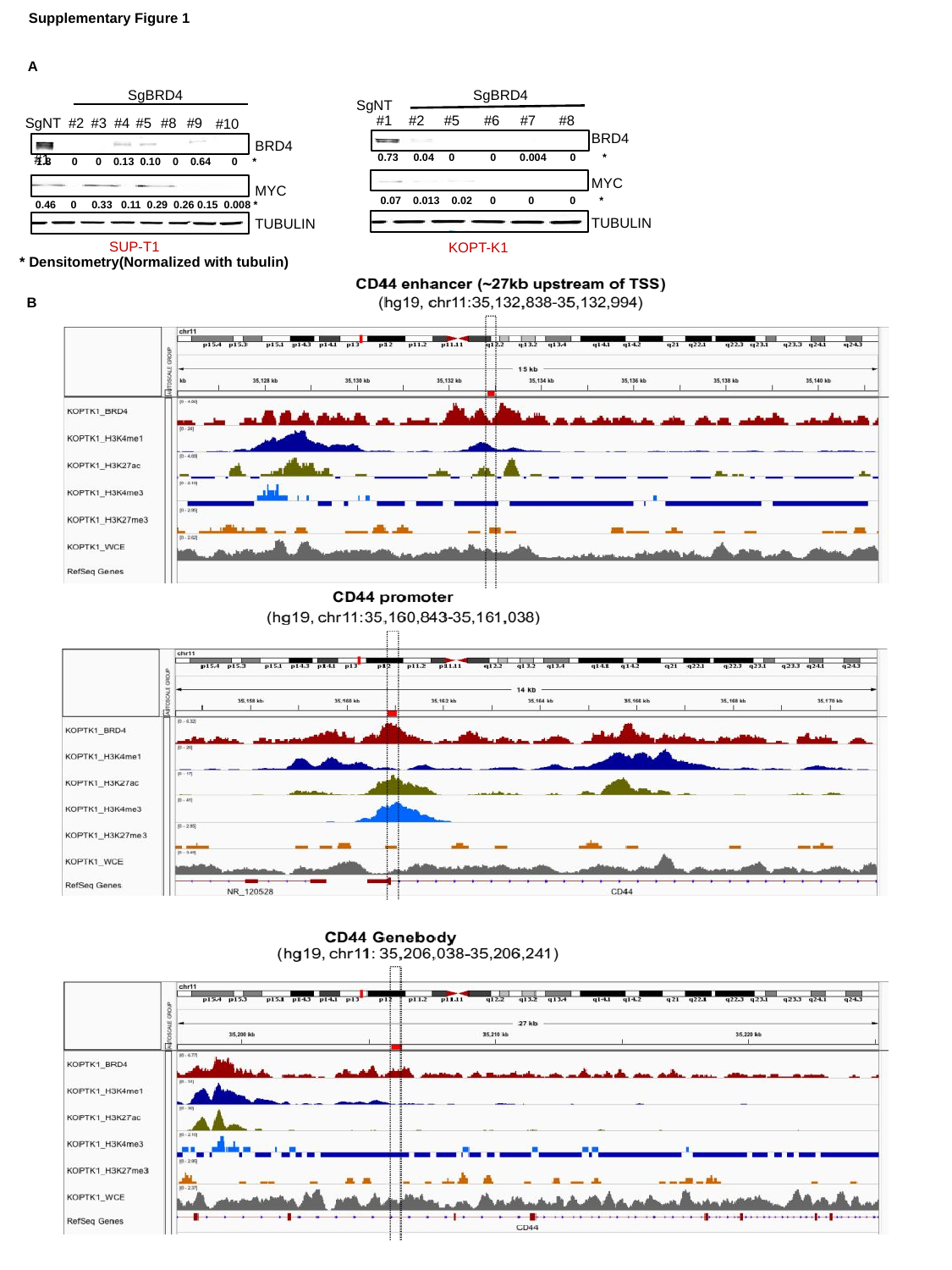

Supplementary Figure 1
A
SgBRD4
SgBRD4
SgNT
#1
#6
#7
#8
#5
#2
#2
#9
#5
#8
#10
#3
#4
BRD4
BRD4
0.73 0.04 0 0 0.004 0 *
1.8 0 0 0.13 0.10 0 0.64 0 *
MYC
MYC
0.07 0.013 0.02 0 0 0 *
0.46 0 0.33 0.11 0.29 0.26 0.15 0.008 *
TUBULIN
SUP-T1
KOPT-K1
* Densitometry(Normalized with tubulin)
TUBULIN
SgNT
#1
B

## Slide 2
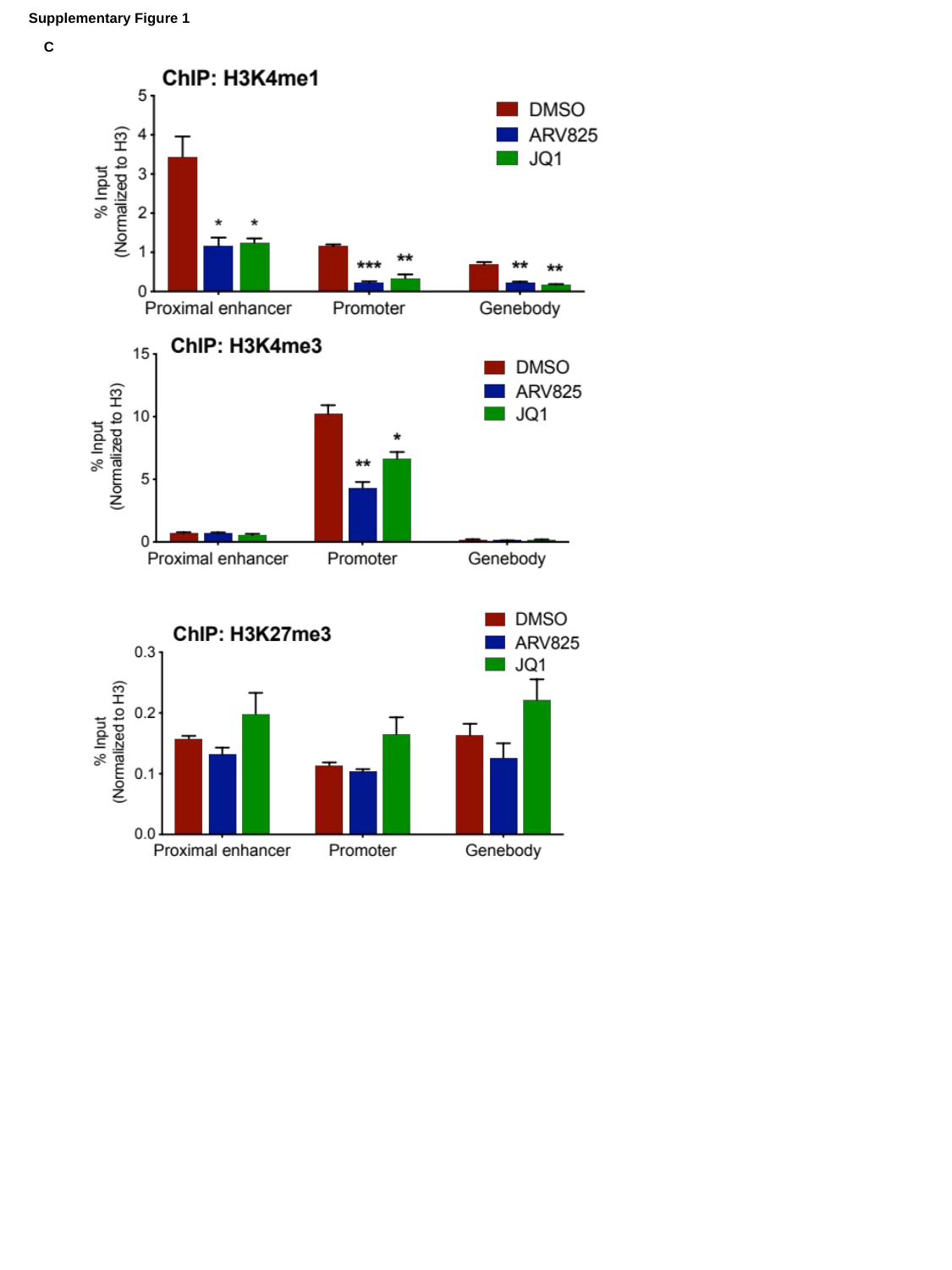

Supplementary Figure 1
C

## Slide 3
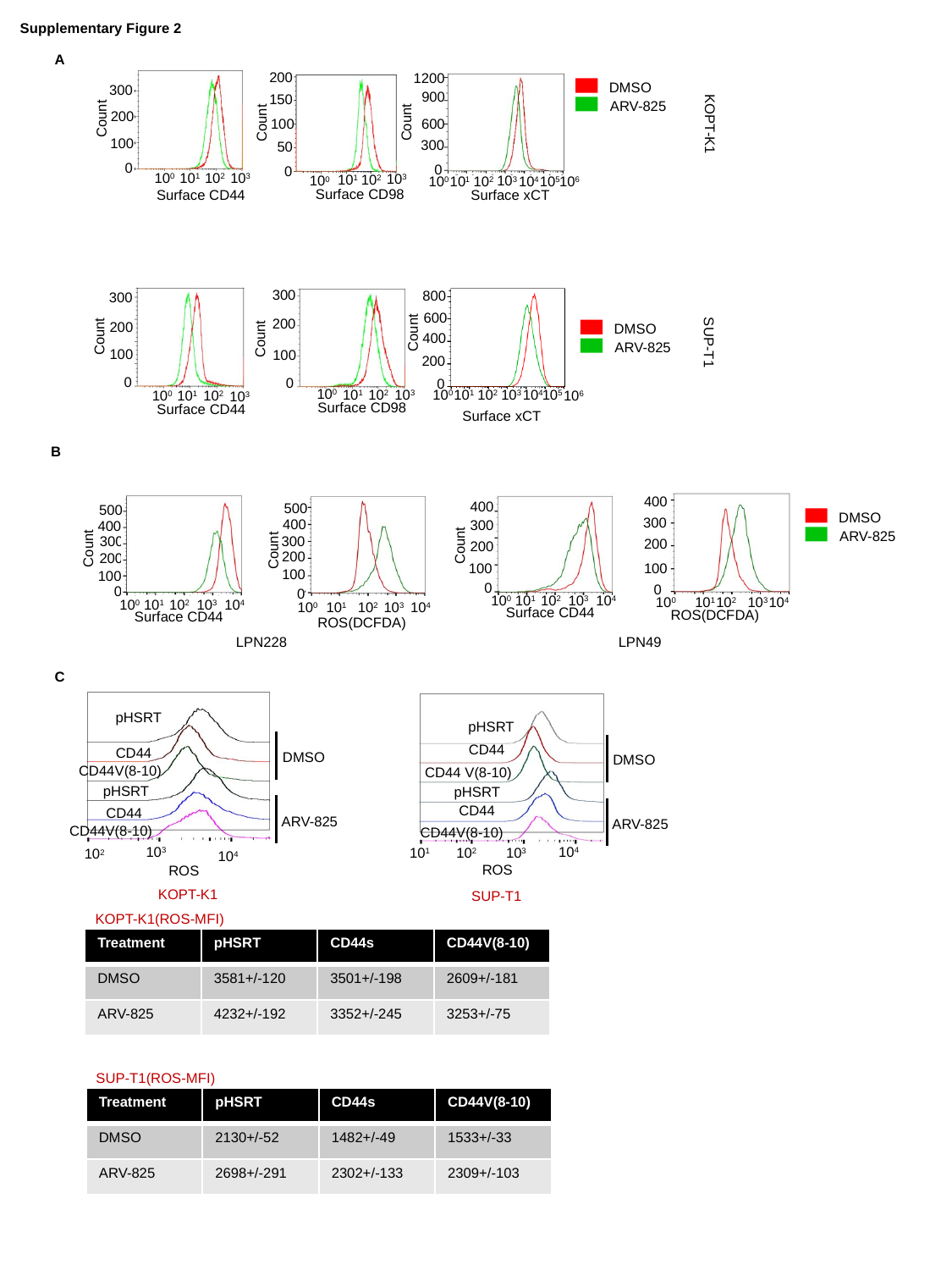

Supplementary Figure 2
A
300
200
100
0
Count
101
102
103
100
Surface CD44
1200
900
600
300
0
Count
103
100
101
102
104
Surface xCT
106
105
200
150
50
0
Count
Surface CD98
100
103
101
102
100
DMSO
ARV-825
KOPT-K1
800
400
200
0
600
Count
103
100
101
102
104
Surface xCT
106
105
300
200
100
0
Count
101
102
100
103
Surface CD44
300
200
100
0
Count
100
101
103
102
Surface CD98
DMSO
ARV-825
SUP-T1
B
300
200
100
0
300
200
100
0
Count
Count
101
102
103
100
104
Surface CD44
101
102
103
100
104
ROS(DCFDA)
LPN228
400
400
300
200
100
0
300
200
100
0
101
102
103
100
104
Surface CD44
101
102
103
100
104
ROS(DCFDA)
LPN49
Count
500
500
400
400
DMSO
ARV-825
C
pHSRT
CD44
pHSRT
CD44
CD44V(8-10)
DMSO
ARV-825
CD44V(8-10)
103
102
104
ROS
KOPT-K1
pHSRT
CD44
pHSRT
CD44
CD44V(8-10)
DMSO
ARV-825
CD44 V(8-10)
104
102
103
ROS
101
SUP-T1
KOPT-K1(ROS-MFI)
| Treatment | pHSRT | CD44s | CD44V(8-10) |
| --- | --- | --- | --- |
| DMSO | 3581+/-120 | 3501+/-198 | 2609+/-181 |
| ARV-825 | 4232+/-192 | 3352+/-245 | 3253+/-75 |
SUP-T1(ROS-MFI)
| Treatment | pHSRT | CD44s | CD44V(8-10) |
| --- | --- | --- | --- |
| DMSO | 2130+/-52 | 1482+/-49 | 1533+/-33 |
| ARV-825 | 2698+/-291 | 2302+/-133 | 2309+/-103 |

## Slide 4
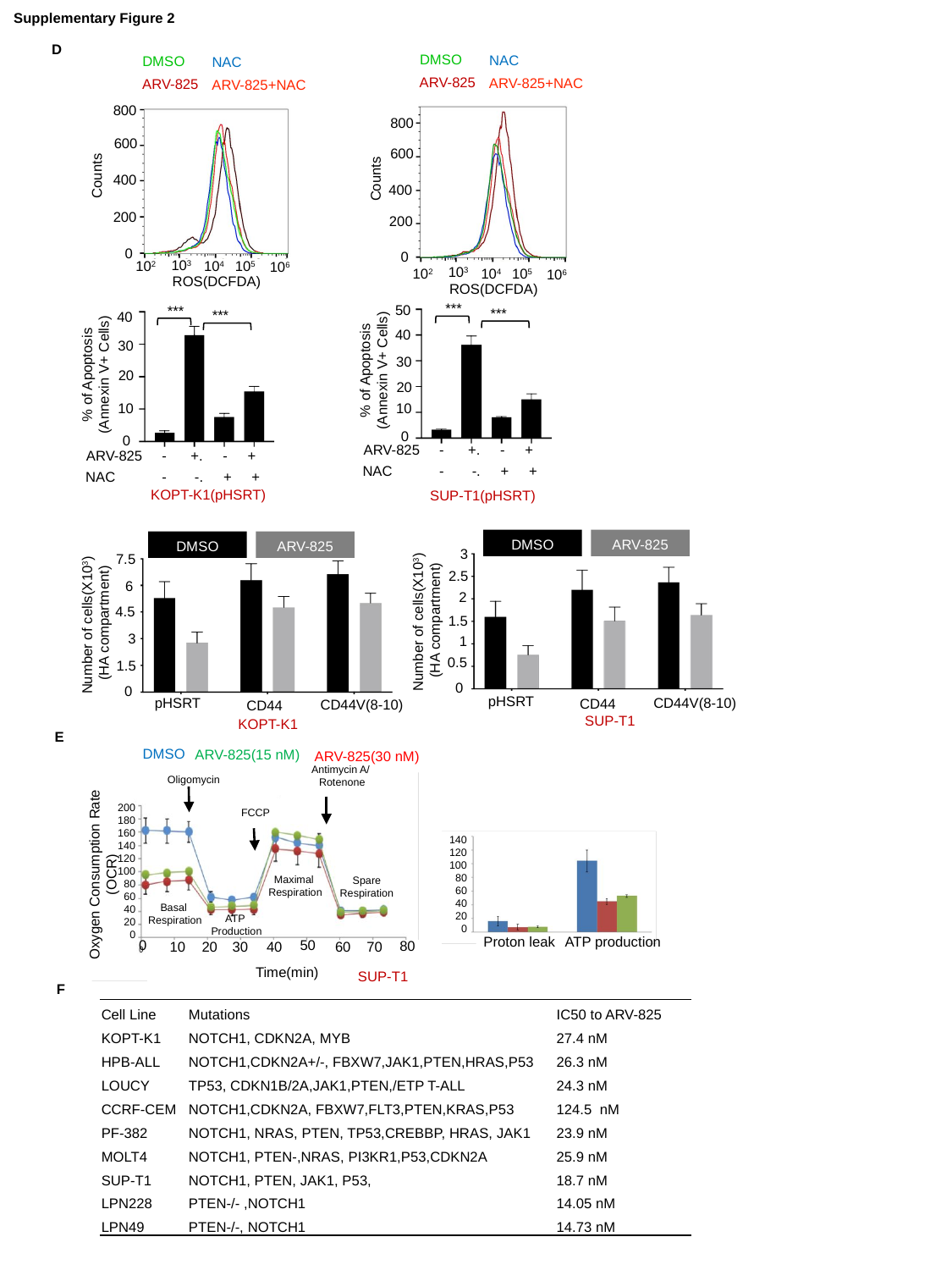

Supplementary Figure 2
D
DMSO
NAC
ARV-825+NAC
ARV-825
800
600
400
0
200
Counts
103
102
104
105
ROS(DCFDA)
106
50
40
30
% of Apoptosis
(Annexin V+ Cells)
20
10
0
ARV-825
- +. - +
NAC
- -. + +
DMSO
NAC
ARV-825+NAC
ARV-825
800
600
400
0
200
Counts
103
102
104
105
ROS(DCFDA)
106
40
30
% of Apoptosis
(Annexin V+ Cells)
20
10
0
ARV-825
- +. - +
NAC
- -. + +
***
***
***
***
KOPT-K1(pHSRT)
SUP-T1(pHSRT)
DMSO
ARV-825
DMSO
ARV-825
3
2
1.5
1
0.5
0
7.5
6
4.5
3
1.5
0
2.5
Number of cells(X103)
(HA compartment)
Number of cells(X103)
(HA compartment)
pHSRT
CD44V(8-10)
pHSRT
CD44
CD44V(8-10)
CD44
SUP-T1
KOPT-K1
E
DMSO
ARV-825(15 nM)
ARV-825(30 nM)
Antimycin A/
RRotenone
FCCP
Maximal
Respiration
Spare
Respiration
Basal
 Respiration
ATP
 Production
Oligomycin
200
180
160
140
120
100
80
60
40
20
0
Oxygen Consumption Rate
(OCR)
0
50
80
30
40
10
20
60
70
Time(min)
140
120
100
80
60
40
20
0
Proton leak
ATP production
SUP-T1
F
| Cell Line | Mutations | IC50 to ARV-825 |
| --- | --- | --- |
| KOPT-K1 | NOTCH1, CDKN2A, MYB | 27.4 nM |
| HPB-ALL | NOTCH1,CDKN2A+/-, FBXW7,JAK1,PTEN,HRAS,P53 | 26.3 nM |
| LOUCY | TP53, CDKN1B/2A,JAK1,PTEN,/ETP T-ALL | 24.3 nM |
| CCRF-CEM | NOTCH1,CDKN2A, FBXW7,FLT3,PTEN,KRAS,P53 | 124.5 nM |
| PF-382 | NOTCH1, NRAS, PTEN, TP53,CREBBP, HRAS, JAK1 | 23.9 nM |
| MOLT4 | NOTCH1, PTEN-,NRAS, PI3KR1,P53,CDKN2A | 25.9 nM |
| SUP-T1 | NOTCH1, PTEN, JAK1, P53, | 18.7 nM |
| LPN228 | PTEN-/- ,NOTCH1 | 14.05 nM |
| LPN49 | PTEN-/-, NOTCH1 | 14.73 nM |

## Slide 5
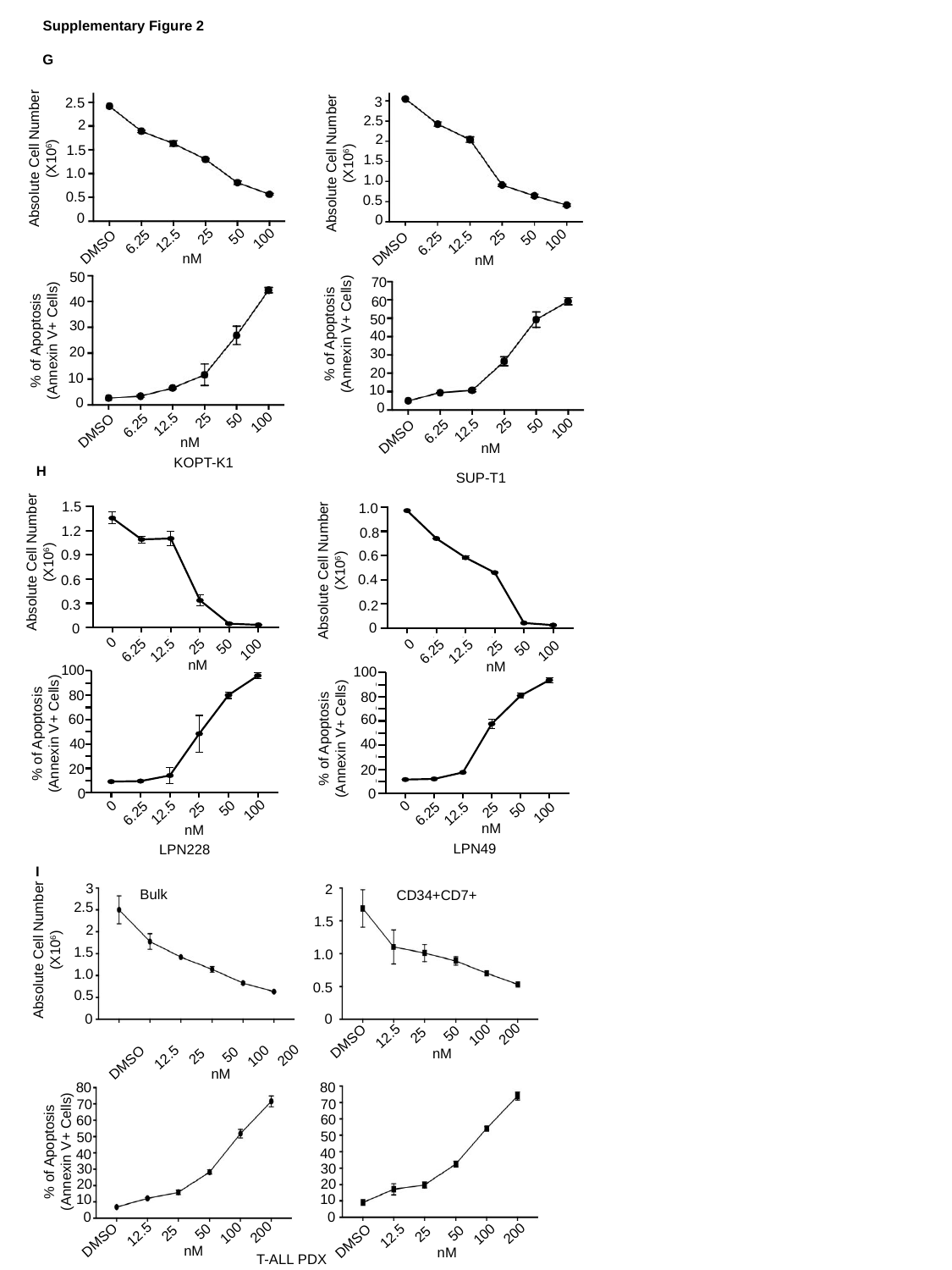

Supplementary Figure 2
G
2.5
2
1.5
1.0
0.5
0
25
50
100
12.5
6.25
DMSO
nM
3
2.5
2
1.5
1.0
0.5
0
25
50
100
12.5
6.25
DMSO
nM
Absolute Cell Number
(X106)
Absolute Cell Number
(X106)
50
40
30
20
10
0
25
50
100
12.5
6.25
DMSO
nM
KOPT-K1
70
60
50
40
30
20
10
0
% of Apoptosis
(Annexin V+ Cells)
% of Apoptosis
(Annexin V+ Cells)
25
50
100
12.5
6.25
DMSO
nM
H
1.5
1.2
0.9
0.6
0.3
0
1.0
0
0.8
Absolute Cell Number
(X106)
0.6
Absolute Cell Number
(X106)
0.4
0.2
0
25
50
100
6.25
12.5
nM
0
25
50
100
6.25
12.5
nM
LPN49
100
80
0
60
40
20
0
25
50
100
6.25
12.5
nM
100
80
0
0
50
100
25
12.5
6.25
LPN228
60
% of Apoptosis
(Annexin V+ Cells)
% of Apoptosis
(Annexin V+ Cells)
40
20
nM
SUP-T1
I
3
2.5
2
1.5
1.0
0.5
0
2
1.5
1.0
0.5
0
Bulk
CD34+CD7+
Absolute Cell Number
(X106)
200
50
100
25
12.5
DMSO
nM
200
50
100
25
12.5
DMSO
nM
80
70
60
50
40
30
20
10
0
80
70
60
50
40
30
20
10
0
% of Apoptosis
(Annexin V+ Cells)
200
50
100
25
12.5
DMSO
nM
200
50
100
25
12.5
DMSO
nM
T-ALL PDX

## Slide 6
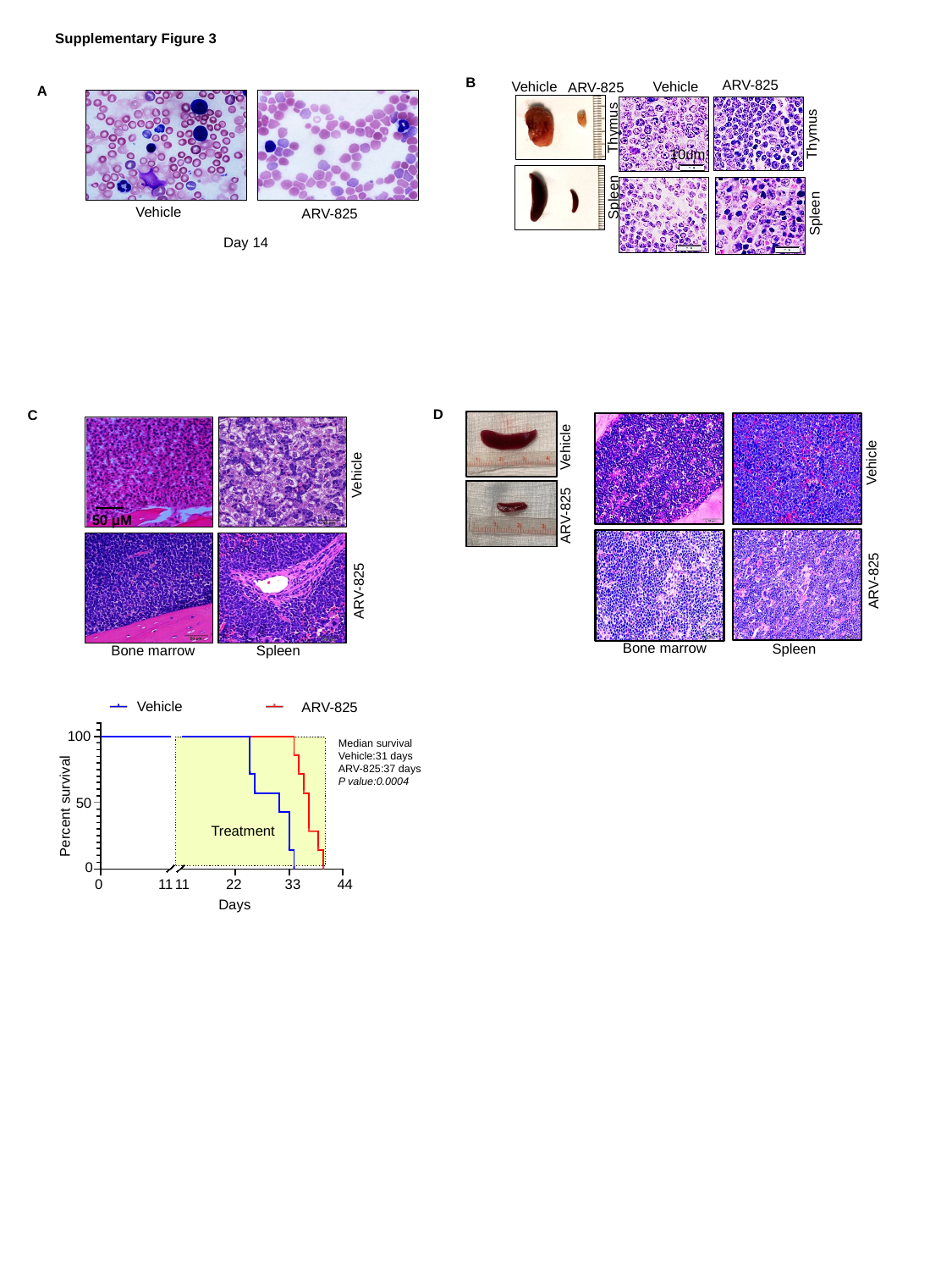

Supplementary Figure 3
B
ARV-825
Vehicle
10um
Thymus
Spleen
Vehicle
ARV-825
Thymus
Spleen
A
Vehicle
ARV-825
Day 14
D
C
Vehicle
Vehicle
ARV-825
ARV-825
Bone marrow
Spleen
Vehicle
ARV-825
Bone marrow
Spleen
50 μM
Vehicle
ARV-825
100
Median survival
Vehicle:31 days
ARV-825:37 days
P value:0.0004
50
Percent survival
Treatment
0
0
11
22
33
44
11
Days

## Slide 7
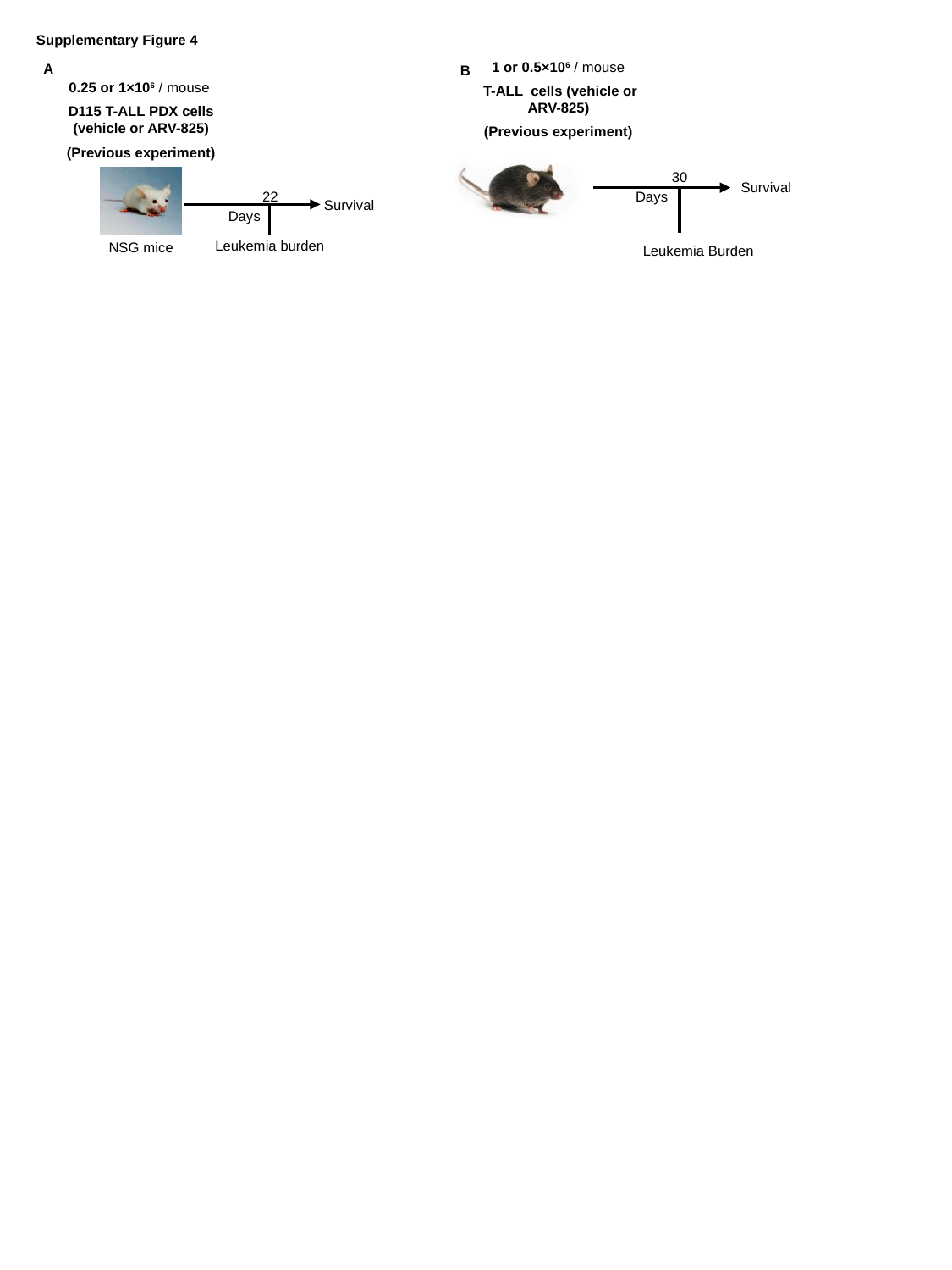

Supplementary Figure 4
 1 or 0.5×10^6^ / mouse
 T-ALL cells (vehicle or ARV-825)
(Previous experiment)
Days
30
Leukemia Burden
Survival
A
B
0.25 or 1×10^6^ / mouse
D115 T-ALL PDX cells (vehicle or ARV-825)
(Previous experiment)
Days
22
Leukemia burden
Survival
NSG mice
