# Supplementary material for: Targeting the NOTCH1-MYC-CD44 axis in leukemia-initiating cells in T-ALL
Source: Leukemia. 2022 Feb 16;36(5):1261–73. doi: 10.1038/s41375-022-01516-1 (PMC9061299; doi:10.1038/s41375-022-01516-1)
Supplement: Supplementary file 3 — Supplementary table [file 41375_2022_1516_MOESM3_ESM.docx]

**Table S1**: Antibodies used in CyTOF for surface and intracellular expression of proteins in T-ALL

| Antigen | Conjugate | Supplier | Catalog number |
| --- | --- | --- | --- |
| ARC 170Er | 170Er | Santa Cruz Biotechnology | sc-374177 |
| Axin2 148Nd | 148Nd | Abcam | ab32197 |
| β-catenin, active | 150Nd | EMD Millipore | 05-665 |
| Bax | 173Yb | Cell Signaling Technology | 2774BF |
| Bcl-2 | 158Gd | BioLegend | 658702 |
| Bcl-xL | 141Pr | Cell Signaling Technology | 2764BF |
| c-Myc | 163Dy | Cell Signaling Technology | 5605BF |
| Caspase-3, cleaved | 142Nd | DVS Sciences | 3142004A |
| CD11b | 173Yb | BioLegend | 301302 |
| CD19 | 161Dy | BioLegend | 302202 |
| CD3 | 170Er | BioLegend | 300443 |
| CD34 | 148Nd | BD | 555820 |
| CD4 | 145Nd | Fluidigm | 3145001B |
| CD44 | 166Er | Fluidigm | 3166001B |
| CD44(Ms/Hu) 171Yb | 171Yb | DVS Sciences | 3171003B |
| CD45 | 89Y | Fluidigm | 3089003B |
| CD7 | 139La | BioLegend | 343102 |
| CD8a | 146Nd | Fluidigm | 3146001B |
| CXCR4 | 172Yb | BioLegend | 306502 |
| Cyclin B1 | 143Nd | BD | 554177 |
| Hes1 | 151Eu | Abcam | ab55265 |
| HEXIM1 161Dy | 161Dy | Proteintech | 15676-1-AP |
| HIF-1a | 159Tb | Novus Biologicals | NB100-479 |
| Ikaros | 144Nd | BioLegend | 653302 |
| Jagged2 166Er | 166Er | BioLegend | 346902 |
| Ki67 | 168Er | Fluidigm | 3168001B |
| Mcl-1 | 176Yb | BD | 559027 |
| mTOR | 162Dy | GenScript | A01154 |
| mTOR 162Dy (MDA) | 162Dy | GenScript | A01154 |
| Notch-1 | 153Eu | BioLegend | 352102 |
| p-AKT 152Sm | 152Sm | Fluidigm | 3152005A |
| p-ERK1/2 | 167Er | DVS Sciences | 3167005A |
| p-FAK 175Lu | 175Lu | Cell Signaling Technology | 8556BF |
| p-MEK1/2 | 169Tm | Cell Signaling Technology | 9154BF |
| p-mTOR | 164Dy | Cell Signaling Technology | 5536BF |
| p-NFκB | 149Sm | Cell Signaling Technology | 3033 |
| p-p38(180/182) | 156Gd | Fluidigm | 3156002A |
| p-PI3K(p85/p55) | 160Gd | Cell Signaling Technology | 4228BF |
| p-STAT5(Y694) | 147Sm | Fluidigm | 3147012A |
| p-SYK | 149Sm | BD | 558167 |
| p21, WAF1/Cip1 | 154Sm | Sigma | P1484 |
| p53 | 165Ho | R&D Systems | MAB1355 |
| RUNX1 | 155Gd | BioLegend | 659302 |

**Table S2:** The primer sequences used in qPCR and ChIP qPCR

| Primer | Forward (5'-3') | Reverse (5'-3') |
| --- | --- | --- |
| CD44 | GCAGTCAACAGTCGAAGAAGG | TGTCCTCCACAGCTCCATT |
| CD44v6 | TCC AGG CAA CTC CTA | CAG CTG TCC CTG TTG |
| CD44v8-10 | TCCCAGACGAAGACAGTCCCTGGAT | CACTGGGGTGGAATGTGTCTTGGTC |
| AXIN2 | CCACACCCTTCTCCAATCC | TGCCAGTTTCTTTGGCTCTT |
| Fra-1 | AGTCAGGAGCTGCAGTGGATGGT | TCAGTTCCTTCCTCCGGTTCCTGC |
| 18S rRNA | GTAACCCGTTGAACCCCATT | CCATCCAATCGGTAGTAGCG |
| hCD44 proximal enhancer | GCAGCACCTCCCTGAAATAA | TGAACCAAGCCAATGTGCTA |
| hCD44 promoter | CGGACACCATGGACAAGTTT | CTTCGCAGACAGCTCACTTG |
| hCD44 gene body | AGGTTGCGTCAGTTTCTGCT | AGCATGTTTCTGTCCCTGCT |

**Table S3**: Details regarding the PDX **cells** used in this study

| Code | Sex | Mutation | Blasts (%) | Experimental figures |
| --- | --- | --- | --- | --- |
| D115 | M | Notch1 mut Q2459^*^, PTEN 245 YQFMFLVW^*^, JAK3 mut | 91 | Figs. 4DEF,5ABCDE, supple 3C |
| 6506870 | M | TET2 mut, U2AF1 mut, WT1 mut | 39 | Fig. 2D, supple 2I |
| CUL76 | N/A | CDKN2A/B mut, Notch1 HD /PEST mut |  | Fig. 4GHI/ suppl 3D |
